# Synthesis and Characterization of Zinc Complexes with Diethyl 2‑Hydroxyazulene-1,3-dicarboxylate as a Ligand

**DOI:** 10.1021/acsomega.6c08016

**Published:** 2026-09-22

**Authors:** Tatsuya Iwashina, Ryoga Yamamoto, Takuya Sagawa, Tomohiro Imai, Kazuki Yamamoto, Takahiro Gunji

**Affiliations:** † Department of Pure and Applied Chemistry, Faculty of Science and Technology, Tokyo University of Science, 2641 Yamazaki, Noda, Chiba 278-8510, Japan; ‡ Department of Industrial Chemistry, Faculty of Engineering, Tokyo University of Science, 6-3-1 Niijuku, Katsushika-ku, Tokyo 125-8585, Japan; § Photocatalysis International Research Center, Tokyo University of Science, 2641 Yamazaki, Noda, Chiba 278-8510, Japan; ∥ Research Group for Advanced Energy Conversion, Research Institute for Science and Technology (RIST), Tokyo University of Science, 2641 Yamazaki, Noda, Chiba 278-8510, Japan

## Abstract

The reaction of diethyl 2-hydroxyazulene-1,3-dicarboxylate (**DEHA**) with zinc species (Zn­(OAc)_2_ and ZnCl_2_) in the presence of various alkali metal carbonates (M_2_CO_3_) was investigated. The resulting complexes were characterized by nuclear magnetic resonance spectroscopy, Fourier-transform infrared spectroscopy, elemental analysis, mass spectrometry, single-crystal X-ray structural analysis, and UV–vis and fluorescence spectroscopy. The reaction between **DEHA** and Zn­(OAc)_2_ in the presence of Rb_2_CO_3_ or K_2_CO_3_ as bases afforded **Zn-DEHA-Rb** or **Zn-DEHA-K**, respectively, where three **DEHA** anions were coordinated to Zn^2+^, with an alkali metal ion as the counter cation. On the other hand, the reaction between **DEHA** and Zn­(OAc)_2_ in the presence of Na_2_CO_3_ afforded **Zn-DEHA-Na-1**, in which four **DEHA** anions were coordinated to two Zn^2+^ and Na^+^, with two acetoxy ions present. The reaction between **DEHA** and ZnCl_2_ in the presence of Na_2_CO_3_ provided **Zn-DEHA-Na-2**, where three **DEHA** anions were coordinated to Zn^2+^, with Na^+^ as the countercation. The zinc complexes exhibited S_1_ emission despite differences in their structures. Furthermore, the synthesized complexes were mixed with poly­(methyl methacrylate) to form composite films.

## Graphical abstract

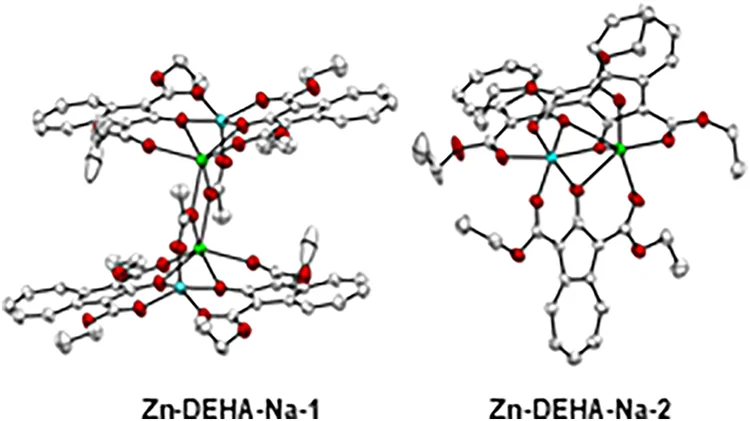

## 1 Introduction

Azulene is a unique molecule, blue in color, and possesses a simple structure.^1^ Azulene is known to exhibit a large dipole moment of 1.08 D^1^ and to display fluorescence from the second excited state in violation of Kasha’s rule.^2^ Its color tone,^3^ electronic state, and solubility can be modulated by introducing substituents into the azulene framework and forming derivatives.

In recent years, several studies have been reported that exploit the unique properties of azulenes in material science.^4–9^ For example, Yamaguchi et al.^9^ developed four isomers of terazulene and applied them as organic field-effect transistor (OFET) materials, thereby enabling control of hole mobility.

Coordination polymers containing azulene moieties are expected to exhibit properties not observed in azulenes alone because of interactions between azulene units and metal species. For example, Sun et al.^10^ reported that a cobalt-coordinated polymer coated on gold rods showed a high rectification effect via the metal-to-ligand transfer transition (MLCT) from Co^2+^ to azulenes. Furthermore, Barman et al.^11^ reported that zinc-based metal–organic frameworks (MOFs) containing azulene derivatives as ligands showed high hydrogen storage capacities owing to the polarity of azulenes. Although coordination polymers containing azulene moieties are expected to exhibit unique properties, the number of reported studies remains limited due to the lack of systematic investigations of metal complexes employing azulenes as ligands, which constitute the fundamental structural units of coordination polymers.

In our previous work, the synthesis and characterization of metal complexes employing diethyl 2-aminoazulene-1,3-dicarboxylate (**DEAA**)^12^ and diethyl 2-hydroxyazulene-1,3-dicarboxylate (**DEHA**)^13^ as ligands were investigated (Scheme S1). **DEAA** formed stable complexes with only Pd^2+^, whereas **DEHA** formed stable complexes with many transition metal ions, including Pd^2+^, Co^2+^, Ni^2+^, Cu^2+^, and Zn^2+^, in the presence of Cs_2_CO_3_ (Figure S1). The Ni^2+^, Co^2+^, and Zn^2+^ complexes with **DEHA** formed coordination polymers containing Cs^+^. Lanthanide complexes with **DEHA** reported by Zhang and Petoud^14^ contained K^+^ ions. Furthermore, the crystal structures of salen^15^ and phenol derivatives^16^ complexes are known to depend on the type of alkali metal ion. Finelli et al. reported that alkali metal complexes utilizing salen-type ligands form discrete molecular structures in the single-crystal state when the metal ion is Li^+^ or Na^+^, whereas they form one-dimensional polymeric structures when the metal ion is K^+^, Rb^+^, or Cs^+^.^15^ Boyle et al. reported that alkali metal complexes with phenol-derivative ligands primarily form discrete molecular structures such as cubes or squares in the single-crystal state for Li^+^ and Na^+^, while primarily forming polymeric structures for K^+^, Rb^+^, and Cs^+^.^16^ Based on these findings, it is known that alkali metal complexes are more likely to form polymeric structures in the single-crystal state as one moves down the periodic table. Therefore, in this study, the structural change in the Zn-**DEHA** complexes was studied by varying the alkali metal carbonate used, as shown in Scheme 1.

**Scheme 1:**
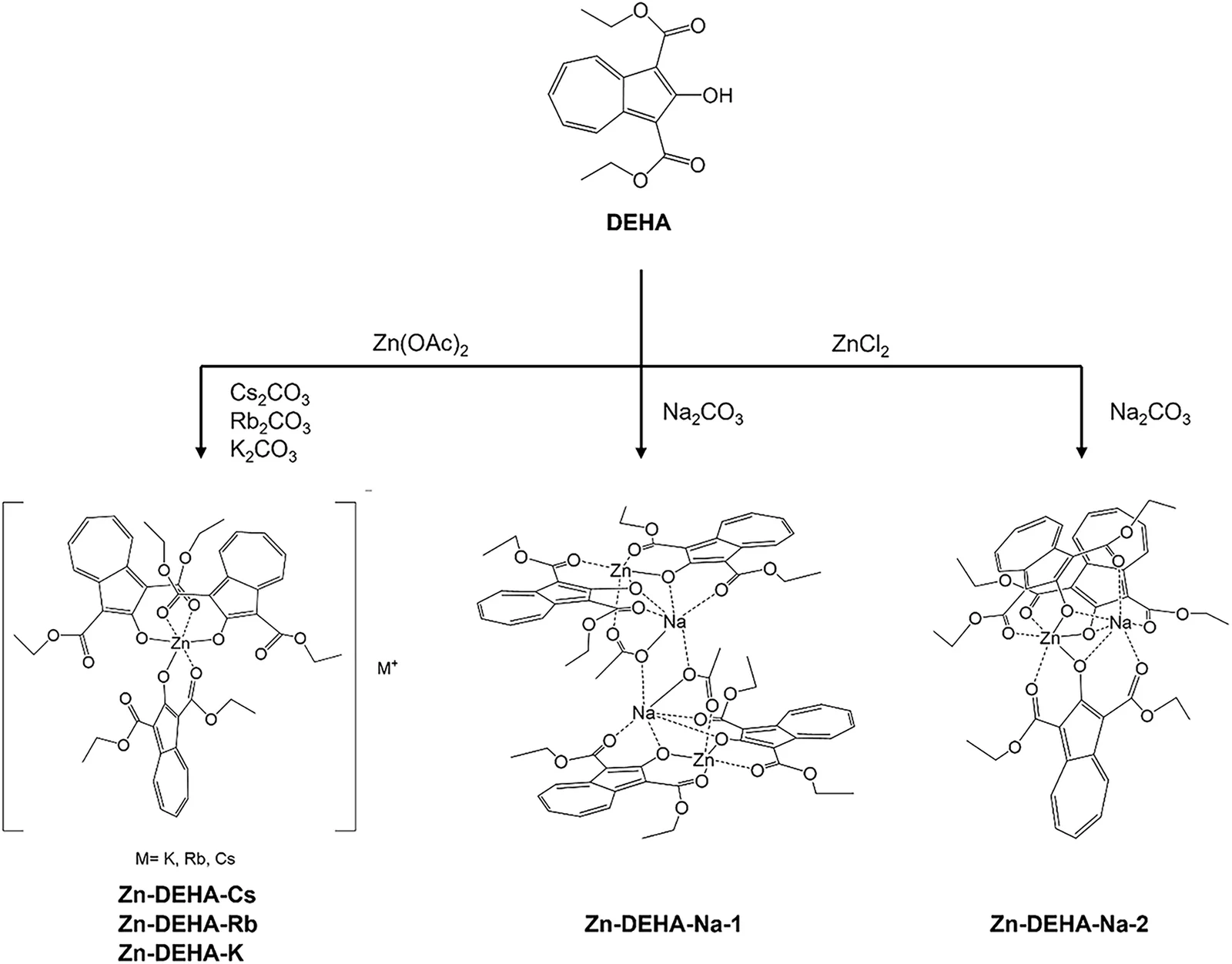
Reactions between **DEHA** and zinc species in the presence of alkali metal carbonates.

## 2 Experimental Section

### 2.1 Characterization

^1^H and ^13^C­{^1^H} nuclear magnetic resonance (NMR) spectra were acquired using a JNM-ECZ400S spectrometer (JEOL, Japan; ^1^H at 399 MHz and ^13^C­{^1^H} at 100 MHz) at 22 ± 5 °C. Chemical shifts of ^1^H and ^13^C­{^1^H} NMR spectra were referenced to the residual proton and carbon signals of the corresponding deuterated solvents, respectively.^17^ Fourier-transform infrared (FTIR) spectroscopy was performed using an FT/IR-6100 spectrometer (JASCO, Japan) equipped with an attenuated total reflectance (ATR) accessory (ZnSe prism, JASCO ATR PRO 0450-S). Mass spectrometry was performed using a JMS-T100CS AccuTOF CS instrument (JEOL, Japan) with electrospray ionization (ESI). Elemental analyses were performed using a UNICUBE elemental analyzer (Elementar, Germany). Thermogravimetric-differential thermal analysis (TG-DTA) was conducted using a TG-DTA 2000SE instrument (Netszch, Japan). The samples were heated to 1000 °C at a rate of 10 °C/min under airflow. The decomposition temperatures were visually recorded using an SMP3 melting point apparatus (Stuart, U.K.). Ultraviolet–visible (UV–Vis) absorption spectra were recorded using a V-670 UV–Vis–NIR spectrophotometer (JASCO, Tokyo, Japan). Single-crystal X-ray diffraction analyses were performed using an XtaLAB Synergy-S diffractometer (RIGAKU, Japan) equipped with a monochromatic Mo–Kα radiation source (λ = 0.7107 Å). Energy-dispersive X-ray spectrometry (EDX) was performed using an SU9000 field-emission scanning electron microscope (Hitachi High-Technologies, Japan) after the samples were coated with gold. Molecular weight estimation from diffusion coefficients obtained by diffusion ordered spectroscopy (DOSY) NMR was performed according to a previously reported method.^18^ The deposition numbers were CCDC 2535569 for **Zn-DEHA-Na-1** and 2535572 for **Zn-DEHA-Na-2**. These data can be obtained free of charge at http://www.ccdc.cam.ac.uk/conts/retrieving.html or from the Cambridge Crystallographic Data Centre, 12 Union Road, Cambridge CB2 1EZ, U.K.; Fax: (+44) 1223–336–033; Email: deposit@ccdc.cam.ac.uk.

### 2.2 Reagents

Hexane, tetrahydrofuran (THF), and sodium hydroxide (NaOH) were purchased from Kanto Chemical Co. (Tokyo, Japan). Cesium carbonate (Cs_2_CO_3_), dichloromethane (CH_2_Cl_2_), sodium carbonate (Na_2_CO_3_), rubidium carbonate (Rb_2_CO_3_), and zinc acetate (Zn­(OAc)_2_) were purchased from FUJIFILM Wako Pure Chemical Corporation (Tokyo, Japan). Zinc chloride (ZnCl_2_) and potassium carbonate (K_2_CO_3_) were purchased from Tokyo Chemical Industry Co., Ltd. (Tokyo, Japan). Chloroform (CHCl_3_) and methanol (CH_3_OH) were purchased from Godo Co., Ltd. (Tokyo, Japan). All solvents were dried over molecular sieves prior to use. A 100 mL vial (AGC Techno Glass Co., Ltd., Shizuoka, Japan) was dried in an oven at 110 °C for >2 h and then cooled in a desiccator before use. Diethyl 2-hydroxyazulene-1,3-dicarboxylate (**DEHA**), **Zn-DEHA-Cs**, and **Na-DEHA** were synthesized as reported previously.^13,19,20^

### 2.3 Synthesis of **Zn-DEHA-Rb** and **Zn-DEHA-K**

In an oven-dried 100 mL vial, **DEHA** (0.10 g, 0.35 mmol), ethanol (EtOH; 30 mL), and Zn­(OAc)_2_ (0.032 g, 0.17 mmol) were combined and dissolved with stirring. Rb_2_CO_3_ or K_2_CO_3_ (0.52 mmol) was then added, and the mixture was stirred at 80 °C for 24 h. The resulting yellow precipitate was collected by filtration and washed with EtOH and then with water. The solid was extracted with CH_2_Cl_2_, and the solvent was removed under reduced pressure. **Zn-DEHA-Rb** and **Zn-DEHA-K** were obtained as orange solids in 68% and 72% yields, respectively, based on **DEHA**.

**Zn-DEHA-Rb:** anal. calcd. for C_48_H_45_O_15_ZnRb (%): C, 56.93; H, 4.48; found (%): C, 56.81; H, 4.51. ESI-HRMS (*m*/*z*): calcd. for [Zn­(DEHA)_3_]^−^ 925.20499; found: 925.20489.

^1^H NMR (399 MHz, THF-*d*_8_, ppm) 1.34 (t, *J* = 7.2 Hz, 18.0 H), 4.33 (q, *J* = 7.2 Hz, 12.0 H), 7.11 (t, *J* = 10 Hz, 3.0 H), 7.30 (t, *J* = 10.0 Hz, 6.0 H), 8.79 (d, *J* = 10.0 Hz, 6.0 H). ^13^C NMR (100 MHz, CD_2_Cl_2_, ppm) 14.8, 60.2, 104.8, 129.4, 130.5, 132.2, 146.3, 168.2, 184.6.

**Zn-DEHA-K:** anal. calcd. for C_48_H_45_O_15_ZnK (%): C, 59.66; H, 4.69; found (%): C, 59.32; H, 4.55. ESI-HRMS (*m*/*z*): calcd. for [Zn­(DEHA)_3_]^−^ 925.20499; found: 925.20499.

^1^H NMR (399 MHz, THF-*d*_8_, ppm) 1.36 (t, *J* = 7.2 Hz, 18.0 H), 4.36 (q, *J* = 7.2 Hz, 12.0 H), 7.12 (t, *J* = 10.0 Hz, 3.0 H), 7.31 (t, *J* = 10.0 Hz, 6.0 H), 8.79 (d, *J* = 10.0 Hz, 6.0 H). ^13^C NMR (100 MHz, CD_2_Cl_2_, ppm) 14.8, 60.3, 104.5, 129.5, 130.6, 132.3, 146.3, 168.3, 184.6.

### 2.4 Synthesis of **Zn-DEHA-Na-1**

In an oven-dried 100 mL vial, **DEHA** (0.10 g, 0.35 mmol), EtOH (30 mL), and Zn­(OAc)_2_ (0.032 g, 0.17 mmol) were combined and dissolved with stirring. Na_2_CO_3_ (0.52 mmol) was added, and the mixture was stirred at 80 °C for 24 h. The yellow precipitate was removed by filtration, and the filtrate was left to stand at room temperature for 2 days. After 2 days, orange crystals that formed were collected by filtration (yield 42% based on Zn­(OAc)_2_) and subjected to single-crystal X-ray structural analysis.

**Zn-DEHA-Na-1:** anal. calcd. for C_68_H_66_O_24_Zn_2_Na_2_ (%): C, 56.56; H, 4.61; found (%): C, 56.17; H, 4.26.

^1^H NMR (399 MHz, THF-*d*_8_, ppm) 1.46 (t, *J* = 7.2 Hz, 24.0 H), 1.81 (s, 6.0 H), 4.49 (q, *J* = 7.2 Hz, 16.0 H), 7.25 (t, *J* = 10.0 Hz, 4.0 H), 7.42 (t, *J* = 10.0 Hz, 8.0 H), 8.89 (d, *J* = 10.0 Hz, 8.0 H). ^13^C NMR (100 MHz, CD_2_Cl_2_, ppm) 14.9, 23.4, 60.8, 104.2, 130.6, 131.6, 133.1, 147.2, 168.9, 177.9, 184.5.

### 2.5 Synthesis of **Zn-DEHA-Na-2**

In an oven-dried 100 mL vial, **DEHA** (0.10 g, 0.35 mmol), EtOH (30 mL), and ZnCl_2_ (0.032 g, 0.17 mmol) were combined and stirred. Na_2_CO_3_ (0.52 mmol) was added, and the mixture was stirred at 80 °C for 24 h. The resulting yellow precipitate was collected by filtration and washed with EtOH, and then with water. The solid was extracted with CH_2_Cl_2_, and the solvent was removed under reduced pressure to afford **Zn-DEHA-Na-2** as an orange solid (yield 46% based on **DEHA**). Single crystals were obtained by slow evaporation from THF-*d*_8_.

**Zn-DEHA-Na-2**: anal. calcd. for C_48_H_45_O_15_ZnNa (%): C, 54.38; H, 4.28; found (%): C, 54.50; H, 4.12. ESI-HRMS (*m*/*z*): calcd. for [Zn­(DEHA)_3_]^−^ 925.20499; found: 925.20484.

^1^H NMR (399 MHz, THF-*d*_8_, ppm) 1.44 (t, *J* = 7.2 Hz, 18.0 H), 4.46 (q, *J* = 7.2 Hz, 12.0 H), 7.16 (t, *J* = 10.0 Hz, 3.0 H), 7.36 (t, *J* = 10.0 Hz, 6.0 H), 8.80 (d, *J* = 10.0 Hz, 6.0 H). ^13^C NMR (100 MHz, THF-*d*_8_, ppm) 14.8, 15.0, 60.0, 105.2, 128.8, 129.6, 132.3, 147.5, 168.5, 186.2.

### 2.6 Preparation of Poly­(methyl methacrylate) (PMMA) Composite Films

PMMA (0.3 g) and **DEHA** or metal**-DEHA** at 0.1, 1, and 3 wt % based on (PMMA) were mixed in THF (5 mL), and the resulting solution was cast into glass Petri dishes with a diameter of 3 cm. The Petri dishes were allowed to stand at room temperature for 1 day and then dried in an electric oven at 80 °C for 1 day.

### 2.7 Computational Methodology

Density functional theory (DFT) and molecular modeling calculations for Azulene, **DEHA**, **Na-DEHA**, and benzo­[*a*]­azulene were performed at the DFT level using Gaussian16 and fully optimized using the B3LYP functional with the 6–31g+ (d,p) basis set.^21–23^ The optimized structures were visualized using GaussView version 6.1.1.^24^

## 3 Results and Discussion

### 3.1 Synthesis and Solubility of Zinc Complexes with DEHA

Zn^2+^ was selected as the transition metal ion because it has no unpaired electrons and is suitable for the NMR analysis. Zn–**DEHA** complexes were synthesized by reacting **DEHA** with Zn­(OAc)_2_ or ZnCl_2_ in the presence of alkali metal carbonate, such as Cs_2_CO_3_, Rb_2_CO_3_, K_2_CO_3_, or Na_2_CO_3_, at a molar ratio of 2:1:3. The elemental compositions of **Zn-DEHA-Rb** and **Zn-DEHA-K** were analyzed by EDX. These analyses confirmed the incorporation of rubidium and potassium cations into **Zn-DEHA-Rb** and **Zn-DEHA-K**, respectively (Figures S1 and S2). The solubilities of Zn**–DEHA** complexes are listed in Table 1. All Zn–**DEHA** complexes were soluble in chloroform and dichloromethane and insoluble in hexane and EtOH. Only **Zn-DEHA-Rb** was insoluble in acetone. For comparison, the solubility of **Na-DEHA** is also included in Table 1; unlike the zinc complexes, **Na-DEHA** was soluble only in THF. Notably, these complexes did not decompose upon washing with water and exhibited resistance to hydrolysis.

**Table 1:** Solubilities of **DEHA** Complexes

	Solubility^a^							
Complex	Hexane	Toluene	Dichloro-methane	Chloroform	THF	Acetone	EtOH	CH_3_OH
**Zn-DEHA-Cs** ^13^	––	–	++	++	++	+	––	–
**Zn-DEHA-Rb**	––	–	+	+	–	––	––	––
**Zn-DEHA-K**	––	–	++	++	++	+	––	–
**Zn-DEHA-Na-1**	––	–	++	++	++	+	––	–
**Zn-DEHA-Na-2**	––	++	++	++	++	++	––	––
**Na-DEHA**	––	––	––	––	++	––	––	––

a Solubility was estimated from the volume of solvent required to dissolve 1 mg of the complex at room temperature. ++: <1 mL, +: 1–5 mL, −: 5–10 mL, −–: >10 mL.

### 3.2 Structure of **Zn-DEHA-Na-1** and **Zn-DEHA-Na-2**

The crystallographic data and selected bond distances obtained from single-crystal X-ray diffraction are summarized in Tables 2 and 3. The acetoxy ions incorporated in **Zn-DEHA-Na-1** were derived from Zn­(OAc)_2_. Single crystals suitable for X-ray analysis could not be obtained for **Zn-DEHA-Rb** or **Zn-DEHA-K**. Based on EDX, elemental analysis, and mass spectrometry, the chemical formulas of **Zn-DEHA-Rb** and **Zn-DEHA-K** were determined to be identical. As a result, the compositions of **Zn-DEHA-Rb** and **Zn-DEHA-K** were determined to be [ZnM­(OC_10_H_5_(COOC_2_H_5_)_2_)_3_] (M = Rb (**Zn-DEHA-Rb**) and K (**Zn-DEHA-K**)) similar to that of **Zn-DEHA-Cs**. The structure of **Zn-DEHA-Na-1** comprises four **DEHA** ligands and two acetoxy ions coordinated to both zinc and sodium ions (Figure 1). The Zn center was five-coordinate, whereas the sodium ion was six-coordinate. Two crystallographically distinct conformations were observed within the **Zn-DEHA-Na-1** crystal, with dihedral angles of 167° (**Zn-DEHA-Na-1** (a)) and 178° (**Zn-DEHA-Na-1** (b)). The Zn–O and Na–O bond lengths were 1.993–2.059 and 2.386–2.485 Å, respectively. τ_5_ values were 0.05 and 0.18, respectively. It was suggested that Zn formed square pyramidal shape rather than trigonal bipyramidal shape in both structures.^25^ In the solid-state packing, an alternating arrangement of the two conformations generated a one-dimensional structure, which was supported by π–π stacking interactions (3.395 Å). These observations suggest that the coexistence of these two conformations facilitated the formation of the one-dimensional assembly.

**Figure 1:**
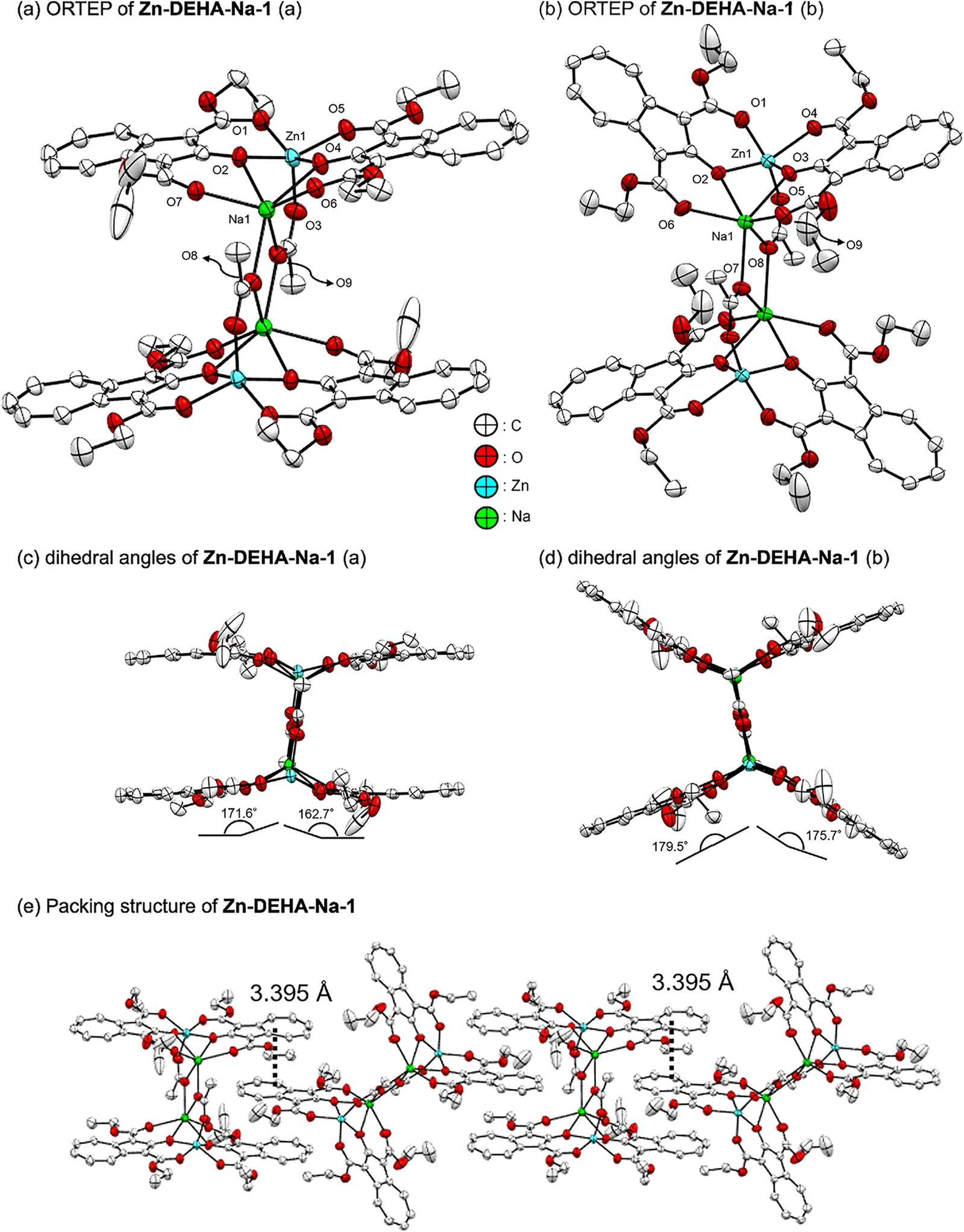
Oak Ridge Thermal Ellipsoid Program (ORTEP) drawing of **Zn-DEHA-Na-1** with the thermal ellipsoids shown at the 50% probability level. Hydrogen atoms are omitted for clarity. (a) ORTEP of **Zn-DEHA-Na-1** (a). (b) ORTEP of **Zn-DEHA-Na-1** (b). (c) dihedral angles of **Zn-DEHA-Na-1** (a). (d) dihedral angles of **Zn-DEHA-Na-1** (b). (e) Packing structure of **Zn-DEHA-Na-1**.

**Table 2:** Selected Bond Distances of **Zn-DEHA-Na-1** (a), **Zn-DEHA-Na-1** (b), and **Zn-DEHA-Na-2**

**Zn-DEHA-Na-1** (a)		**Zn-DEHA-Na-1** (b)		**Zn-DEHA-Na-2**	
Bond	Distance (Å)	Bond	Distance (Å)	Bond	Distance (Å)
Zn1–O1	2.059(3)	Zn1–O1	2.046(2)	Zn1–O1	2.090(2)
Zn1–O2	1.996(2)	Zn1–O2	2.003(2)	Zn1–O2	2.088(2)
Zn1–O3	1.965(2)	Zn1–O3	1.993(3)	Zn1–O3	2.074(2)
Zn1–O4	1.995(2)	Zn1–O4	2.077(2)	Zn1–O4	2.044(2)
Zn1–O5	2.050(2)	Zn1–O5	1.942(2)	Zn1–O5	2.027(2)
Na1–O2	2.439(3)	Na1–O2	2.413(3)	Zn1–O6	2.059(2)
Na1–O7	2.393(3)	Na1–O3	2.386(2)	Na1–O4	2.325(2)
Na1–O8	2.495(3)	Na1–O6	2.436(2)	Na1–O5	2.443(2)
Na1–O9	2.459(2)	Na1–O7	2.418(2)	Na1–O6	2.437(2)
Na1–O6	2.450(3)	Na1–O8	2.485(2)	Na1–O7	2.406(2)
Na1–O4	2.399(2)	Na1–O9	2.394(3)	Na1–O8	2.340(2)
				Na1–O9	2.405(2)

**Table 3:** Crystallographic Data of **Zn-DEHA-Na-1** and **Zn-DEHA-Na-2**

Compound	**Zn-DEHA-Na-1**	**Zn-DEHA-Na-2**
CCDC	2535569	2535572
Empirical formula	[0.5·C_68_H_66_O_24_Na_2_ZN_2_]	[C_48_H_45_O_15_NaZn·2(C_4_D_8_O)]
Formula weight	721.96	1010.50
Space group	*P*1̅	*P*1̅
Crystal system	triclinic	triclinic
*a* (Å)	12.0992 (2)	13.0052(6)
*b* (Å)	13.1700(2)	14.7033(5)
*c* (Å)	20.9736(4)	15.4734(6)
α (°)	86.6020(10)	86.425(3)
β (°)	76.133(2)	75.274(4)
γ (°)	86.2200(10)	67.279(4)
*Z*	4	2
Temp. (K)	93.90(10)	99
*F* (000)	1496	1148
*V* (Å^3^)	3234.34	2637.4
*d* (g/cm^3^)	1.483	1.398
μ (mm^–1^)	0.838	0.547
Reflection collected	71871	62531
Independent reflections	16462	13497
(*R*_int_)	0.1335	0.0690
*R*_1_ (*I* > 2σ*(I)*)	0.0598	0.0576
*w*R**_2_ (all data)	0.1488	0.1680
Goodness of Fit	0.878	1.177

**Zn-DEHA-Na-2** consisted of three **DEHA** ligands coordinated to zinc and sodium ions, similar to **Zn-DEHA-Cs** (Figure 2). This structural difference from **Zn-DEHA-Na-1** reflects the use of ZnCl_2_ as the zinc source, which does not introduce acetoxy ion. Two THF-*d*_8_ molecules were present per **Zn-DEHA-Na-2** unit as crystallization molecules. Both zinc and sodium were six-coordinate. The Zn–O and Na–O bond lengths were 2.027–2.090 and 2.325–2.443 Å, respectively, and the average dihedral angle was 171°. τ_6_ value was 0.24. It was suggested that Zn formed octahedron shape rather than trigonal prismatic shape.^26^ On the other hand, τ_6_ values of **Zn-DEHA-Cs** was 0.13 and the structure around Zn formed octahedron similarly to **Zn-DEHA-Na-2**. In the packing structure, π–π stacking interactions were observed (3.367 Å). However, unlike **Zn-DEHA-Cs**, **Zn-DEHA-Na-2** did not form a one-dimensional coordination polymer, which may be attributed to the difference in ionic radii between the sodium and cesium ions. For (troponato)­sodium­(I) hydride (Na­(Tp)·H_2_O), the Na–O distances were 2.326 and 2.318 Å (CCDC: 1217301).^27^ For (2,4-pentanedionato)­sodium­(I) (Na­(acac)), the Na–O distances were 2.367 and 2.447 Å (CCDC: 119051).^28^ For hexa­(2,4-pentanedionato)­trizinc­(II) (Zn_3_(acac)_6_), the Zn–O distances were 1.971–2.167 Å (CCDC: 1313743).^29^ Accordingly, the Na–O and Zn–O bond lengths in **Zn-DEHA-Na-1** and **Zn-DEHA-Na-2** are comparable to those of Na­(Tp)·H_2_O, Na­(acac), and Zn_3_(acac)_6_.

**Figure 2:**
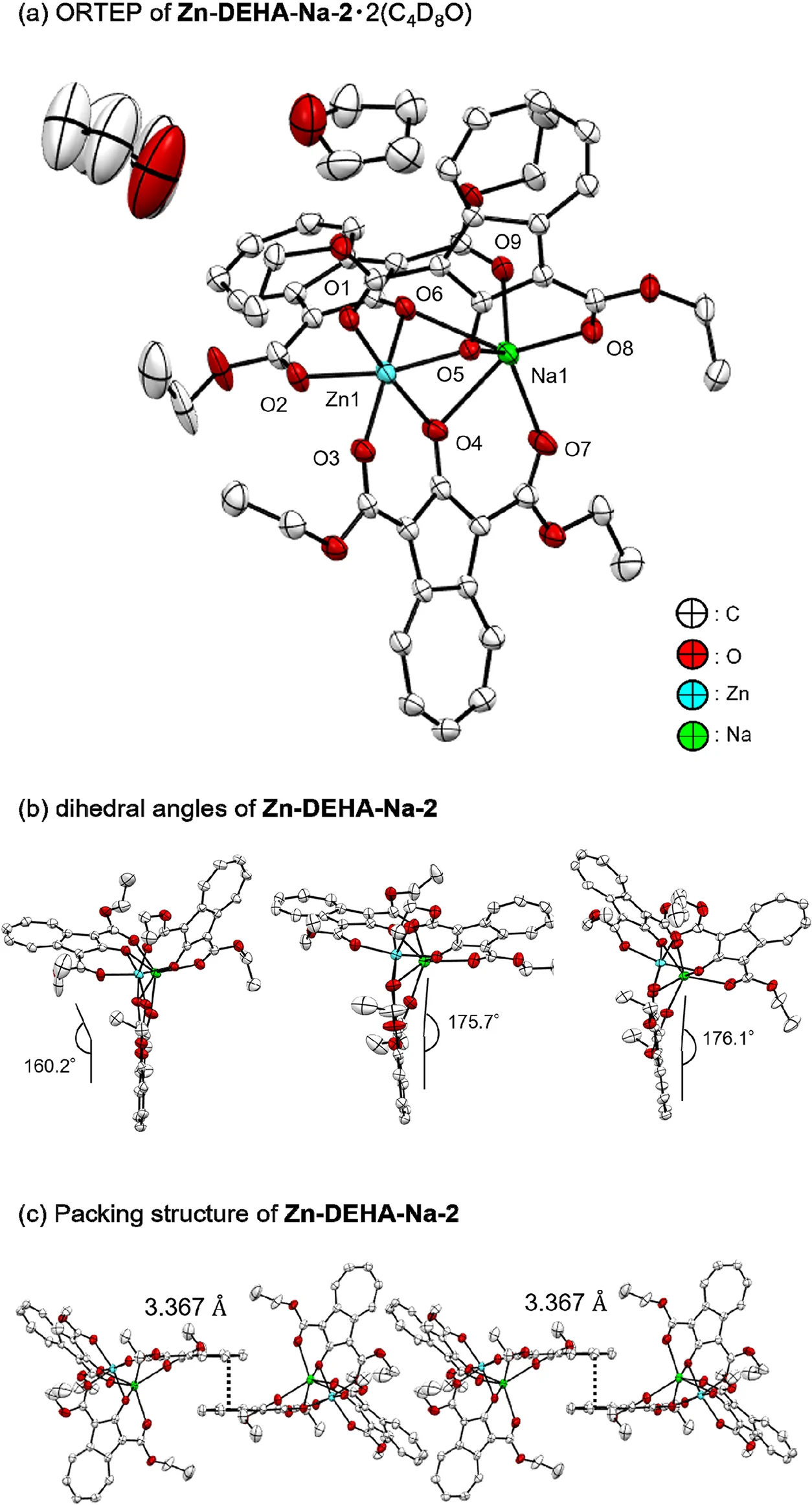
ORTEP drawing of **Zn-DEHA-Na-2** with the thermal ellipsoids shown at the 50% probability level. Hydrogen atoms are omitted for clarity. (a) ORTEP drawing of **Zn-DEHA-Na-2**·2­(C_4_D_8_O). (b) Dihedral angles of **Zn-DEHA-Na-2**. (c) Packing structure of **Zn-DEHA-Na-2**.

### 3.3 Spectra of **DEHA** Complexes

The Zn-**DEHA** complexes were characterized by ^1^H NMR and FTIR spectroscopy. The ^1^H NMR spectra of the Zn-**DEHA** complexes are shown in Figure 3, together with the spectrum of **Na-DEHA** for comparison. For all **DEHA** complexes, the signal attributable to the hydroxy proton of **DEHA** (11.5 ppm) was absent, consistent with complexation. In addition, the signals of the azulenyl moieties shifted to higher magnetic fields relative to those of **DEHA**, as reported for **Pd-DEHA** and **Zn-DEHA-Cs**.^13^ The ethoxy-group quartet signals of **Zn-DEHA-Cs**, **Zn-DEHA-Rb**, **Zn-DEHA-K**, and **Na-DEHA** also shifted upfield, whereas those of **Zn-DEHA-Na-1** and **Zn-DEHA-Na-2** shifted downfield. These results indicate that, among complexes with the same metal-to-ligand ratio (**Zn-DEHA-Cs**, **Zn-DEHA-Rb**, **Zn-DEHA-K**, and **Zn-DEHA-Na-2**), Cs^+^, Rb^+^, and K^+^ behave as relatively electron-rich cations, whereas Na^+^ behaves as a relatively electron-deficient cation. For sodium-containing complexes (**Zn-DEHA-Na-1**, **Zn-DEHA-Na-2**, and **Na-DEHA**), the direction and magnitude of the ethoxy quartet shift depended on whether zinc or acetoxy ions were present. DOSY NMR was performed to further investigate the solution-state structures. The molecular weights of **DEHA** and metal-**DEHA** complexes estimated from the diffusion coefficients are summarized in Table 4. Based on the molecular weights estimated in THF-*d*_8_, **Zn-DEHA-Cs**, **Zn-DEHA-Rb**, **Zn-DEHA-Na-1**, and **Na-DEHA** were inferred to form oligomeric species, whereas **Zn-DEHA-K** and **Zn-DEHA-Na-2** were inferred to be predominantly monomeric. These results suggest that the upfield shift observed in **Zn-DEHA-Cs** and **Zn-DEHA-Rb** can be attributed to the synergistic effect of electron-rich Cs^+^ and Rb^+^ ions and oligomerization. **Na-DEHA** was estimated to exist as a monomer in DMSO-*d*_6_.

**Figure 3:**
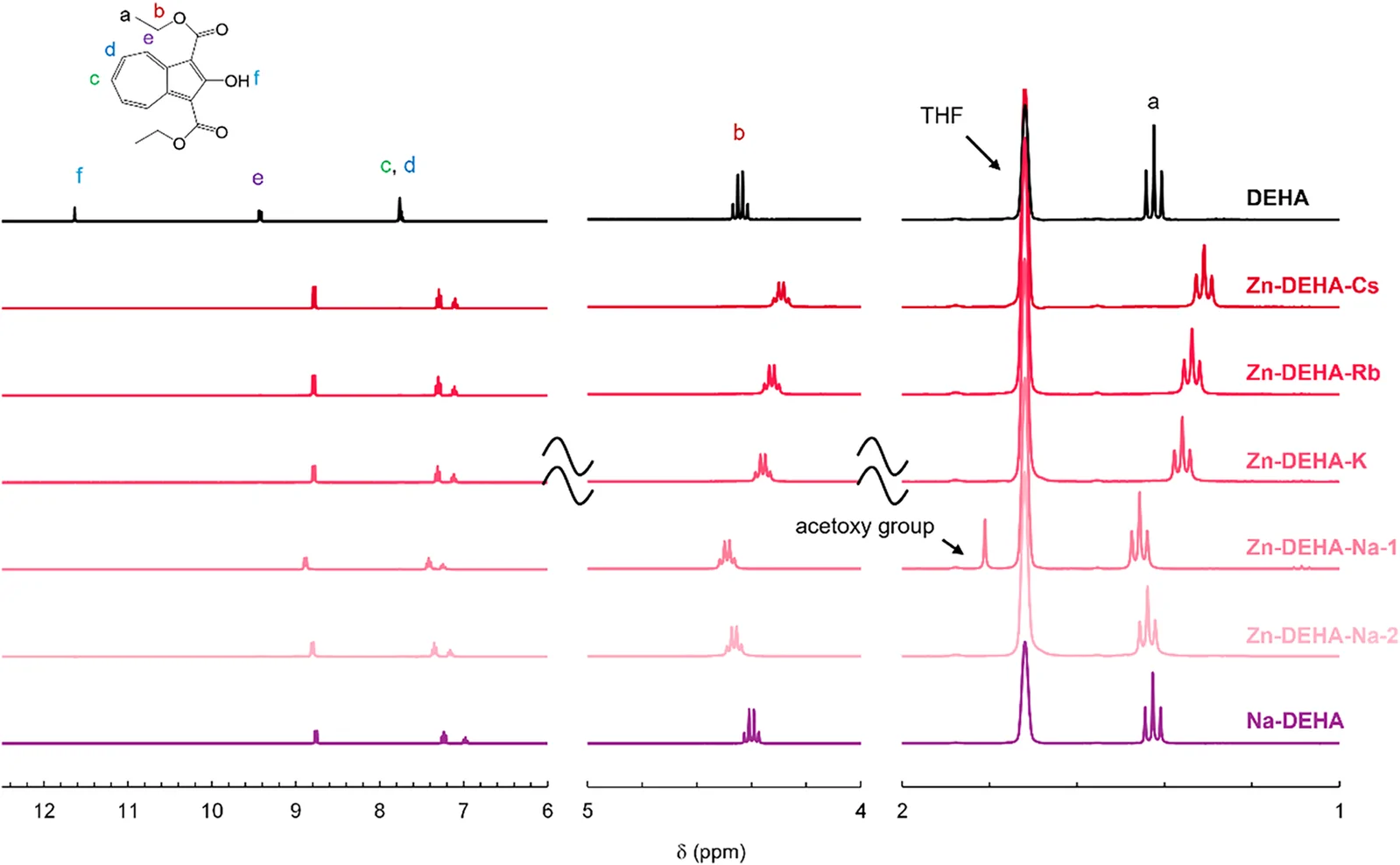
^1^H NMR spectra of **DEHA**, Zn-**DEHA** complexes, and **Na-DEHA** recorded in THF-*d*_8_.

**Table 4:** Molecular Weights of **DEHA** and Metal-**DEHA** Complexes

		Molecular weight estimated by DOSY NMR	
Compound	Molecular weight	In THF-*d*_8_	In DMSO-*d*_6_
**DEHA**	287	36	236
**Zn-DEHA-Cs**	1058	331	-^a^
**Zn-DEHA-Rb**	1010	173	-
**Zn-DEHA-K**	964	101	-
**Zn-DEHA-Na-1**	1440	590	-
**Zn-DEHA-Na-2**	948	108	-
**Na-DEHA**	330	134	330

a –, not applicable.

The FTIR spectra of **DEHA** and **DEHA** complexes are shown in Figure 4, and the corresponding assignments are summarized in Table 5. The absorption band of **DEHA** at 1673 cm^–1^ was assigned to υ­(C=O) vibration of free C=O groups. The absorption bands of the Zn-**DEHA** complexes at 1666–1683 cm^–1^ were assigned to the υ­(C=O) vibration of C=O groups coordinated to alkali metal ions. In the single-crystal structures, the C=O bond lengths for carbonyl groups coordinated to Na were 1.203–1.222 Å in **Zn-DEHA-Na-1** and 1.212–1.222 Å in **Zn-DEHA-Na-2**; the corresponding distances for carbonyl groups coordinated to Cs were 1.198–1.214 Å in **Zn-DEHA-Cs**. These values did not substantially deviate from those of free C=O bonds (1.211 Å in **DEHA** and 1.217 Å in **Pd-DEHA**). Accordingly, only minor differences were observed in the FTIR spectra between the υ­(C=O) bands of free C=O groups and those of C=O groups coordinated to alkali metal ions. Consistent with this interpretation, **Na-DEHA** exhibited a band at 1660 cm^–1^, which was assigned to υ­(C=O) of C=O coordinated to the sodium ion. In contrast, the absorption bands of the Zn-**DEHA** complexes at 1590–1606 cm^–1^ were assigned to υ­(C=O) of C=O coordinated to the zinc ion.

**Figure 4:**
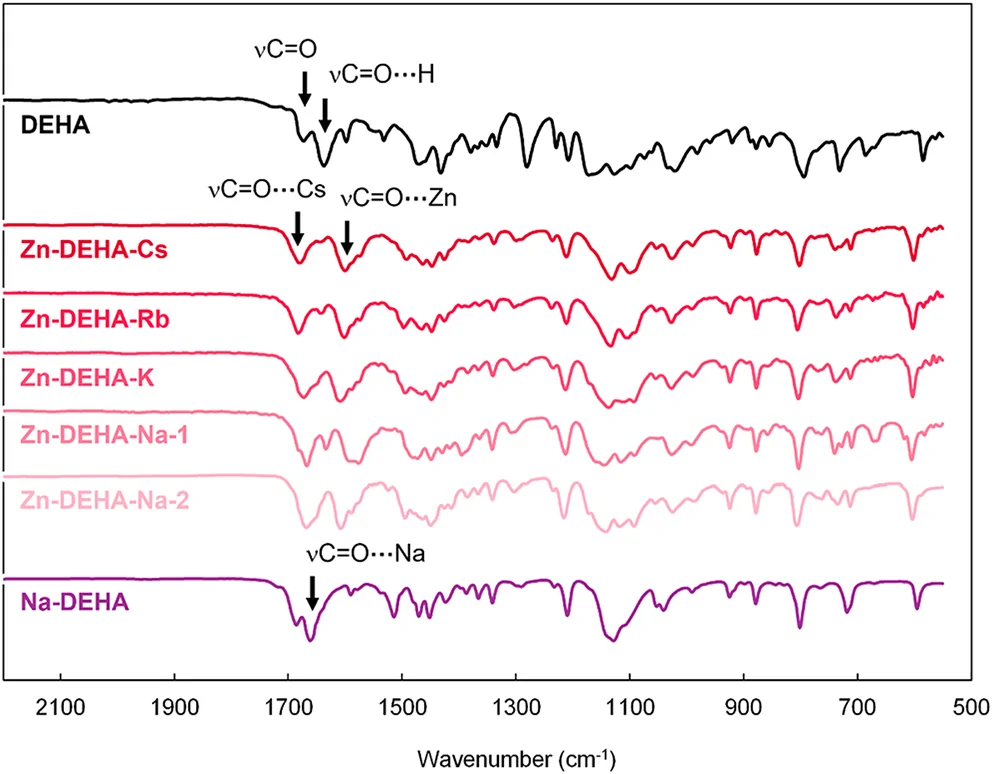
FTIR spectra of **DEHA** and the **DEHA** complexes recorded using the ATR method.

**Table 5:** FTIR Assignments of **DEHA** and the **DEHA** Complexes

Wavenumber (cm^–1^)							
**DEHA**	**Zn-DEHA-Cs**	**Zn-DEHA-Rb**	**Zn-DEHA-K**	**Zn-DEHA-Na-1**	**Zn-DEHA-Na-2**	**Na-DEHA**	Assignment^a^
1673						1685	υ(C=O) of free C=O
	1677	1683	1671	1666	1668	1660	υ(C=O) of C=O coordinated to alkali metal ion
1639							υ(C=O) of hydrogen bond
	1600	1601	1608	1590	1606		υ(C=O) of C=O coordinated to zinc ion

a υ, stretching.

The UV–Vis spectra of **DEHA** and the Zn-**DEHA** complexes are shown in Figure 5. The absorption band of **DEHA** at 430 nm, assigned to the S_0_ → S_1_ transition, red-shifted to 455–459 nm for the Zn-**DEHA** complexes. The absorption band of **DEHA** at 363 nm, assigned to the S_0_ → S_2_ transition, red-shifted to 393–404 nm for the Zn-**DEHA** complexes. The absorption band of **DEHA** at 313 nm, assigned to the S_0_ → S_3_ transition, red-shifted to 331–332 nm for the Zn-**DEHA** complexes. Overall, the absorption features of the Zn-**DEHA** complexes were similar to those reported for other metal-**DEHA** complexes.^13,14^ No significant shifts in UV–Vis spectra were observed due to differences in metal species and coordination structures. Furthermore, since significant change by complexation was not observed in the planar structure of **DEHA**, the absorption of these complexes was thought to be attributable to **DEHA**. DOSY NMR results indicated that **Zn-DEHA-Cs**, **Zn-DEHA-Rb**, and **Zn-DEHA-Na-1** formed oligomers while **Zn-DEHA-K** and **Zn-DEHA-Na-2** formed monomers. Therefore, π–π stacking due to proximity of the ligands may be formed in **Zn-DEHA-Na-1** and **Zn-DEHA-Cs** which formed higher-order oligomers. On the other hand, π–π stacking was not formed in **Zn-DEHA-K** and **Zn-DEHA-Na-2** because of forming monomer like single crystal structure (Figure 2). In UV–Vis spectra, there was no significant shifts between oligomers (**Zn-DEHA-Cs** and **Zn-DEHA-Na-1**) and monomers (**Zn-DEHA-K** and **Zn-DEHA-Na-2**). Hence, in UV–Vis spectra of **Zn-DEHA-Cs** and **Zn-DEHA-Na-1**, it was suggested that no π–π interactions existed or a weak π–π interactions existed.^30^

**Figure 5:**
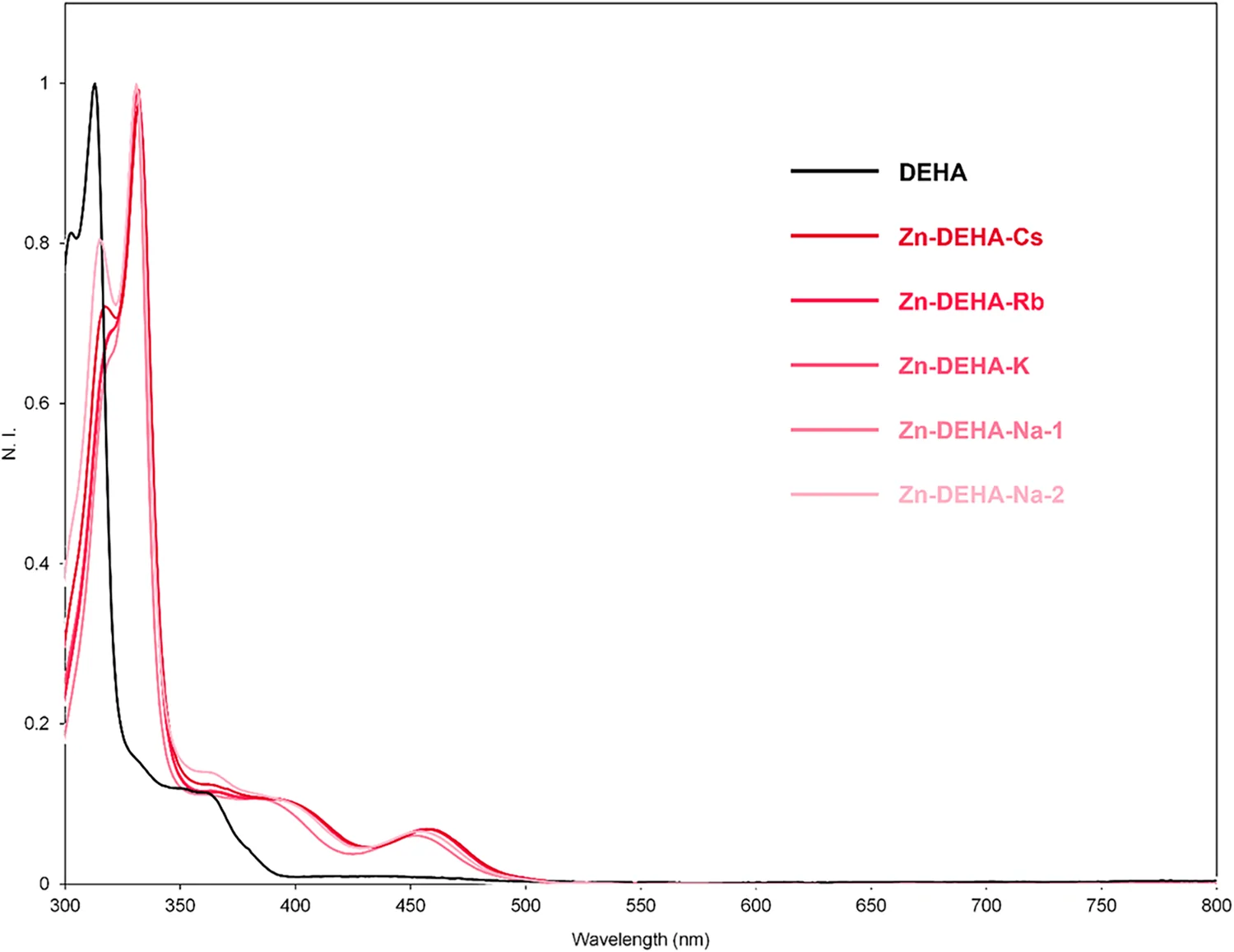
UV–Vis spectra of **DEHA** and the Zn-**DEHA** complexes in CH_2_Cl_2_ (10^–5^ mol/L).

The fluorescence spectra of **DEHA** and the corresponding complexes are shown in Figure 6. In our previous work,^13^ the fluorescence of **DEHA** was assigned to the S_2_ emission. In contrast, all zinc complexes exhibited S_1_ emission, as observed for **Zn-DEHA-Cs**, regardless of structural differences and types of alkali metal ions. The reason for this behavior was further examined using Time-Dependent Density Functional Theory (TD-DFT) calculations. Due to the high computational cost associated with the large size of the zinc complexes, TD-DFT calculations were not performed on the full systems. Therefore, **Na-DEHA** was selected as a simplified model to investigate the excited-state properties and it displays S_1_ emission similar to that of the zinc complexes. Furthermore, benzo­[*a*]­azulene,^31^ which exhibits S_2_ emission, was used as a reference system to compare a heterometallacycle with a fused benzene ring. Recently, Dunlop et al.^32^ attributed the S_2_ emission of azulene to excited-state aromaticity. Specifically, they proposed that low aromaticity in S_1_ promotes thermal deactivation, whereas higher aromaticity in S_2_ favors radiative decay. We applied this framework to our system. Aromaticity was evaluated according to the harmonic oscillator model of aromaticity (HOMA), using the optimized structure from the TD-DFT calculation. The S_0_, S_1_, and S_2_ structures of azulene, **DEHA**, **Na-DEHA**, and benzo­[*a*]­azulene were optimized using DFT at the B3LYP/6–31g+ (d,p) level. The optimized structures of **DEHA** and **Na-DEHA** are shown in Figures S3 and S4, respectively. In **DEHA**, the carbonyl group not engaged in hydrogen bonding was rotated by 24° relative to the azulene ring in the S_1_ structure. In **Na-DEHA**, the carbonyl group not coordinated to the sodium ion was rotated by 44° relative to the azulene ring in the S_1_ and S_2_ structures. The TD-DFT-derived HOMA values for S_0_, S_1_, and S_2_ in azulene, **DEHA**, **Na-DEHA**, and benzo­[*a*]­azulene are summarized in Table 6. For azulene and **DEHA**, the HOMA values decreased from 0.96 to 0.87 and from 0.89 to 0.78, respectively, when going from S_2_ to S_1_, consistent with their S_2_ emission. Conversely, **Na-DEHA** and benzo­[*a*]­azulene showed no substantial change in the HOMA values between S_2_ and S_1_, which accounts for the S_1_ emission of **Na-DEHA** but does not explain the S_2_ emission of benzo­[*a*]­azulene. To explain the S_2_ emission of benzo­[*a*]­azulene, the S_2_–S_1_ energy gap was considered. In azulene derivatives, fluorescence is generally dominated by S_2_ emission when the S_2_–S_1_ gap exceeds 10,000 cm^–1^, whereas both S_2_ and S_1_ emissions can coexist when the S_2_–S_1_ gap is 9000–10,000 cm^–1^.^33^ When the S_2_–S_1_ gap is below 9000 cm^–1^, S_1_ emissions dominates.^34^ The S_2_–S_1_ gaps of azulene, **DEHA**, **Na-DEHA**, and benzo­[*a*]­azulene calculated by TD-DFT are summarized in Table 6. The S_2_–S_1_ gap of benzo­[*a*]­azulene was 10,015 cm^–1^, thereby supporting its S_2_ emission. Conversely, the S_2_–S_1_ gap of **Na-DEHA** was calculated to be 0 cm^–1^, well below 9000 cm^–1^. Thus, the S_1_ emission of **Na-DEHA** can be explained by both the small change in excited-state aromaticity and the small S_2_–S_1_ gap. Collectively, these results suggest that complexation shifts **DEHA** fluorescence from S_2_ to S_1_ emission by minimizing differences in aromaticity between S_2_ and S_1_ and by reducing the S_2_–S_1_ gap to below 9000 cm^–1^. However, it should be noted that the S_1_ emission of these complexes could not be distinguished from the S_1_ and S_2_ emissions based on the calculation results.

**Figure 6:**
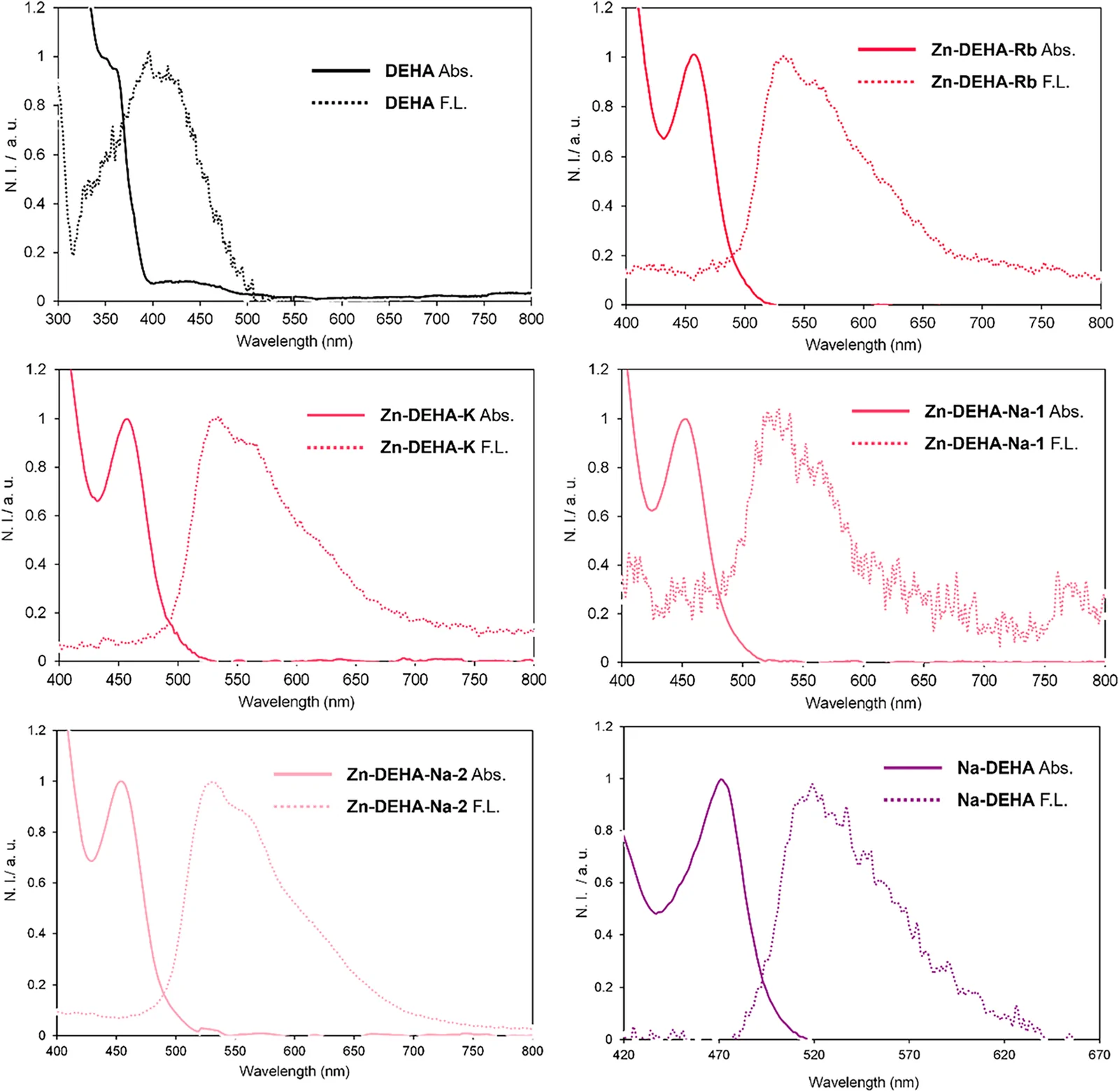
Fluorescence spectra of **DEHA** and the Zn-**DEHA** complexes in CH_2_Cl_2_ and **Na-DEHA** in THF (λ_em_ = 310 nm for **DEHA**; λ_em_ = 330 nm for the metal-**DEHA** complexes).

**Table 6:** HOMA and S_2_–S_1_ Gap Values

Compound	S_0_	S_1_	S_2_	S_2_–S_1_ gap (cm^–1^)
azulene	1	0.87	0.96	11,953
**DEHA**	0.97	0.78	0.89	5337
**Na-DEHA**	0.89	0.74	0.74	0
benzo[*a*]azulene	0.91	0.88	0.89	10,015

### 3.4 PMMA Composite Films Containing **DEHA** Complexes

PMMA composite films containing 0.1, 1, and 3 wt % of **Zn-DEHA-Rb**, **Zn-DEHA-K**, **Zn-DEHA-Na-1**, **Zn-DEHA-Na-2**, and **Na-DEHA** were prepared for application as dyes dispersed in polymers. The appearance and UV–Vis spectra of the PMMA composite films are shown in Figure S5 and Figures S6–S10, respectively. In the UV–vis spectra, only the PMMA composite films containing **Zn-DEHA-K** exhibited a high transmittance of approximately 90% at loadings up to 3 wt %, whereas the transmittance of films containing other complexes decreased with increasing complex content. The dispersion estimated from the UV–vis spectra showed a weaker correlation with the solubility of the metal-**DEHA** complexes in THF than that reported in previous studies.^13^ This weaker correlation is thought to result from the loss of stabilization by THF coordination during drying in the composite film formation process. In the FTIR spectra, no shifts were observed in the PMMA^31^ carbonyl band around 1730 cm^–1^ (Figure S11). The composite films exhibited improved thermal stability, as reflected by generally higher 20% weight-loss temperatures (*T*_d20_) compared with pure PMMA (Figures S12–S16). *T*_d20_ values of PMMA-metal-**DEHA** complexes are summarized in Table 7. This improvement is thought to be due to the interaction between PMMA and metal-**DEHA**. However, the absence of significant shifts in the IR spectra (Figure S11) and the lack of substantial improvement in *T*_d10_ ^35^ suggest that there is no major interaction between PMMA and metal-**DEHA**.

**Table 7:** *T*_d20_ Values of PMMA-Metal-DEHA Composite Films

Complex	Content (wt %)	*T*_d20_ (°C)
PMMA only	-^a^	287
**Zn-DEHA-Cs**	3	327
	1	327
	0.1	308
**Zn-DEHA-Rb**	3	337
	1	321
	0.1	289
**Zn-DEHA-K**	3	330
	1	329
	0.1	303
**Zn-DEHA-Na-1**	3	325
	1	340
	0.1	288
**Zn-DEHA-Na-2**	3	332
	1	326
	0.1	317
**Na-DEHA**	3	318
	1	324
	0.1	320

a –, not applicable.

## 4 Conclusion

The reactions between **DEHA** and zinc species (Zn­(OAc)_2_ and ZnCl_2_) in the presence of alkali metal carbonates (Rb_2_CO_3_, K_2_CO_3_, and Na_2_CO_3_) were investigated. The reaction of **DEHA** with Zn­(OAc)_2_ in the presence of Rb_2_CO_3_ or K_2_CO_3_ afforded [ZnM­(OC_10_H_5_(COOC_2_H_5_)_2_)_3_] (M = Rb (**Zn-DEHA-Rb**) and K (**Zn-DEHA-K**)). In contrast, the reaction of **DEHA** with Zn­(OAc)_2_ and Na_2_CO_3_ yielded [Zn_2_Na_2_(OC_10_H_5_(COOC_2_H_5_)_2_)_4_(CH_3_COO)_2_] (**Zn-DEHA-Na-1**). In **Zn-DEHA-Na-1**, the acetoxy ion derived from Zn­(OAc)_2_ was present, as confirmed by ^1^H NMR, ^13^C NMR, elemental analysis, and single-crystal X-ray diffraction. The single-crystal structure revealed two conformations that differed in the azulene-ring angle, enabling π–π interactions. When **DEHA** was reacted with ZnCl_2_ and Na_2_CO_3_, [ZnNa­(OC_10_H_5_(COOC_2_H_5_)_2_)_3_] (**Zn-DEHA-Na-2**) was formed. Unlike **Zn-DEHA-Na-1**, **Zn-DEHA-Na-2** contained no acetoxy ions. Moreover, **Zn-DEHA-Na-2** did not form a coordination polymer, in contrast to **Zn–DEHA–Cs**, which may be attributable to the difference in ionic radii between Cs^+^ and Na^+^. In the ^1^H NMR spectra, the signals of the azulene moiety in all Zn-**DEHA** complexes shifted upfield relative to those of **DEHA**. The ethoxy-group resonances shifted either upfield or downfield depending on the alkali metal ion. In the FTIR spectra, the υ­(C=O) band to C=O groups coordinated to the zinc ion shifted to lower wavenumbers relative to the υ­(C=O) band associated with hydrogen-bonded carbonyl groups. Furthermore, the carbonyl stretching frequencies showed no significant change between the υ­(C=O) band of free C=O groups and that of C=O groups coordinated to alkali metal ions. In the fluorescence spectra, the S_2_ emission of **DEHA** shifted to S_1_ emission upon complexation with zinc and sodium ions. This shift was attributed to the small difference in aromaticity between S_1_ and S_2_ and the small S_2_–S_1_ gap. PMMA-metal-**DEHA** composite films were prepared, and their heat resistance was evaluated by TG-DTA. The results showed improved heat resistance compared with pure PMMA. These results suggest that the Zn-**DEHA** complexes can be used as building blocks for functional materials such as MOFs incorporating Zn-**DEHA** units.

## Supplementary Material







## Notes

20231019 Zn-DEHA-Na-1 (CIF)
20250725 Zn-DEHA-Na-2 (CIF)
Experimental details, IR, UV–Vis, EDX, TG-DTA traces, and DFT calculations (PDF)
