# Supplementary material for: Synthesis and Characterization of Zinc Complexes with Diethyl 2‑Hydroxyazulene-1,3-dicarboxylate as a Ligand
Source: ACS Omega. 2026 Sep 22;11(39):60289–99. doi: 10.1021/acsomega.6c08016 (PMC13648081; doi:10.1021/acsomega.6c08016)
Supplement: Supplementary file 1 [file ao6c08016_si_003.pdf]

Supporting Information

Synthesis and Characterization of Zinc Complexes with Diethyl 2-Hydroxyazulene-1,3-dicarboxylate as a Ligand

Tatsuya Iwashina[1,*], Ryoga Yamamoto[1], Takuya Sagawa[2], Tomohiro Imai[1], Kazuki Yamamoto[1], Takahiro Gunji[1,3,*]

[1] Department of Pure and Applied Chemistry, Faculty of Science and Technology, Tokyo University of Science, 2641 Yamazaki, Noda, Chiba 278-8510, Japan.

[2] Department of Industrial Chemistry, Faculty of Engineering, Tokyo University of Science, 6-3-1 Niijuku, Katsushika-ku, Tokyo, 125-8585, Japan

[3] Photocatalysis International Research Center, Tokyo University of Science, 2641 Yamazaki, Noda, Chiba 278-8510, Japan.

* Corresponding authors' E-mail addresses: 7221511@alumni.tus.ac.jp (T. Iwashina), gunji@rs.tus.ac.jp (T. Gunji)

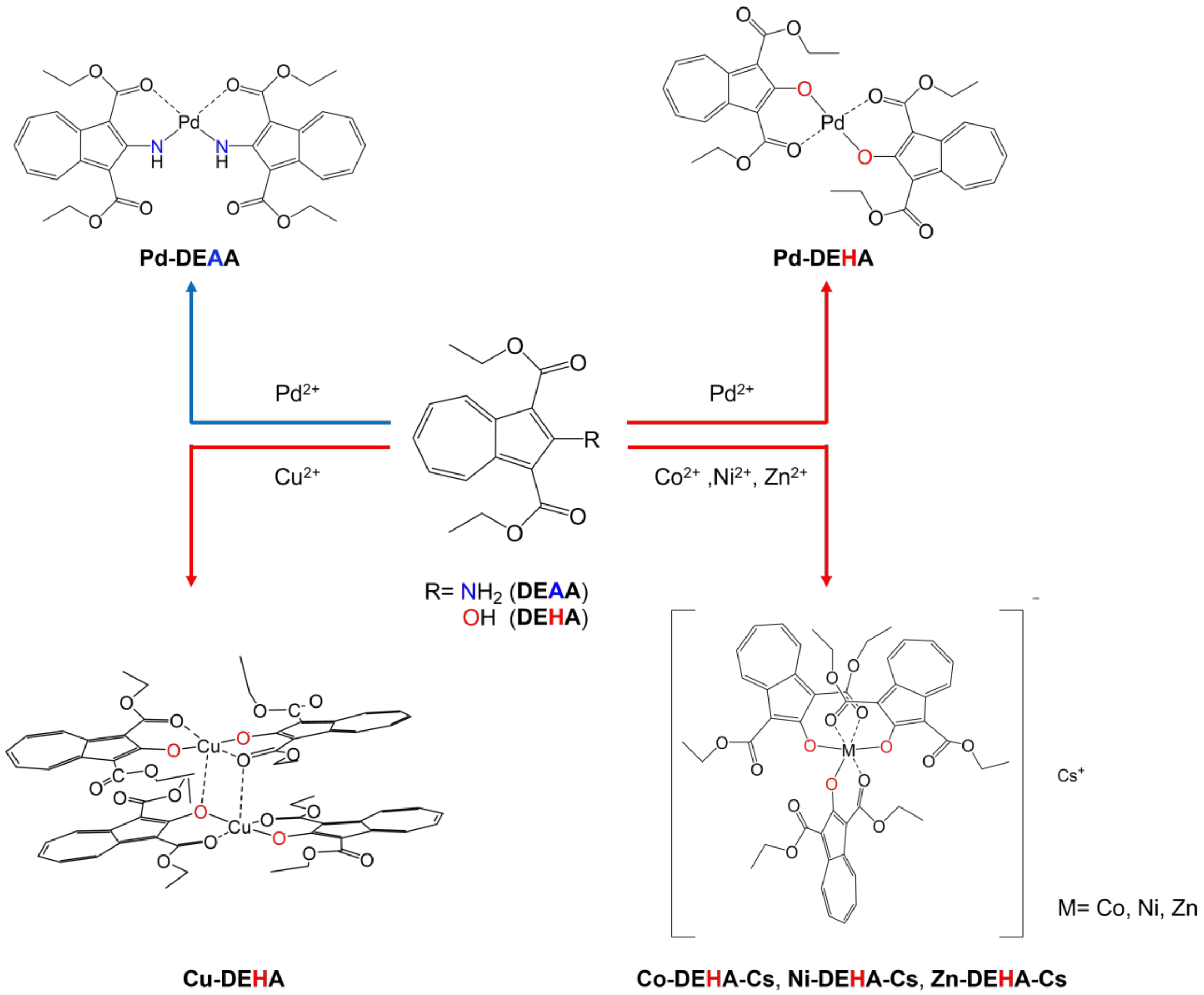

Scheme S1. Synthesis of **DEAA** and **DEHA** complexes.

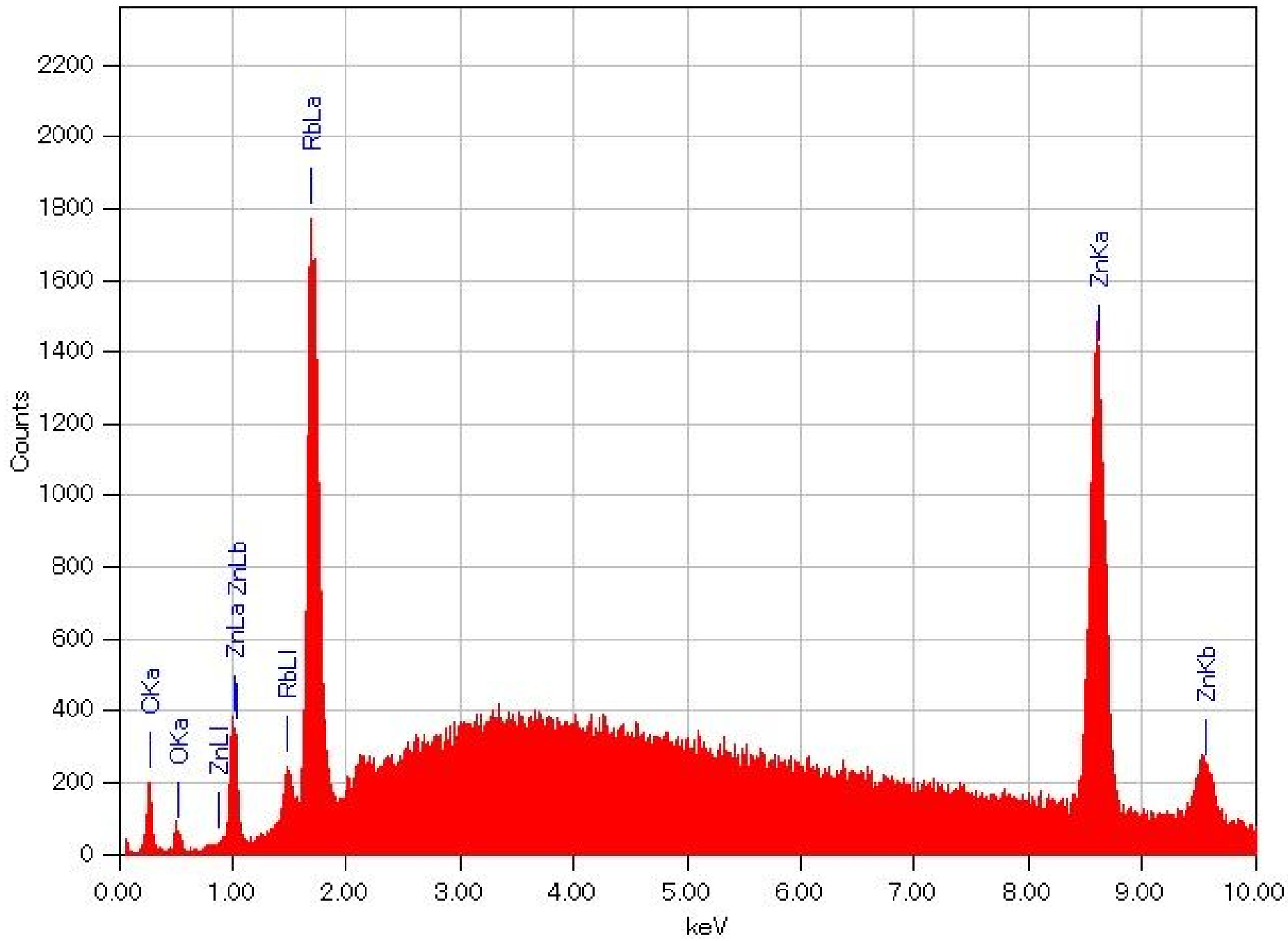

Figure S1. EDX spectra of **Zn-DEHA-Rb**.

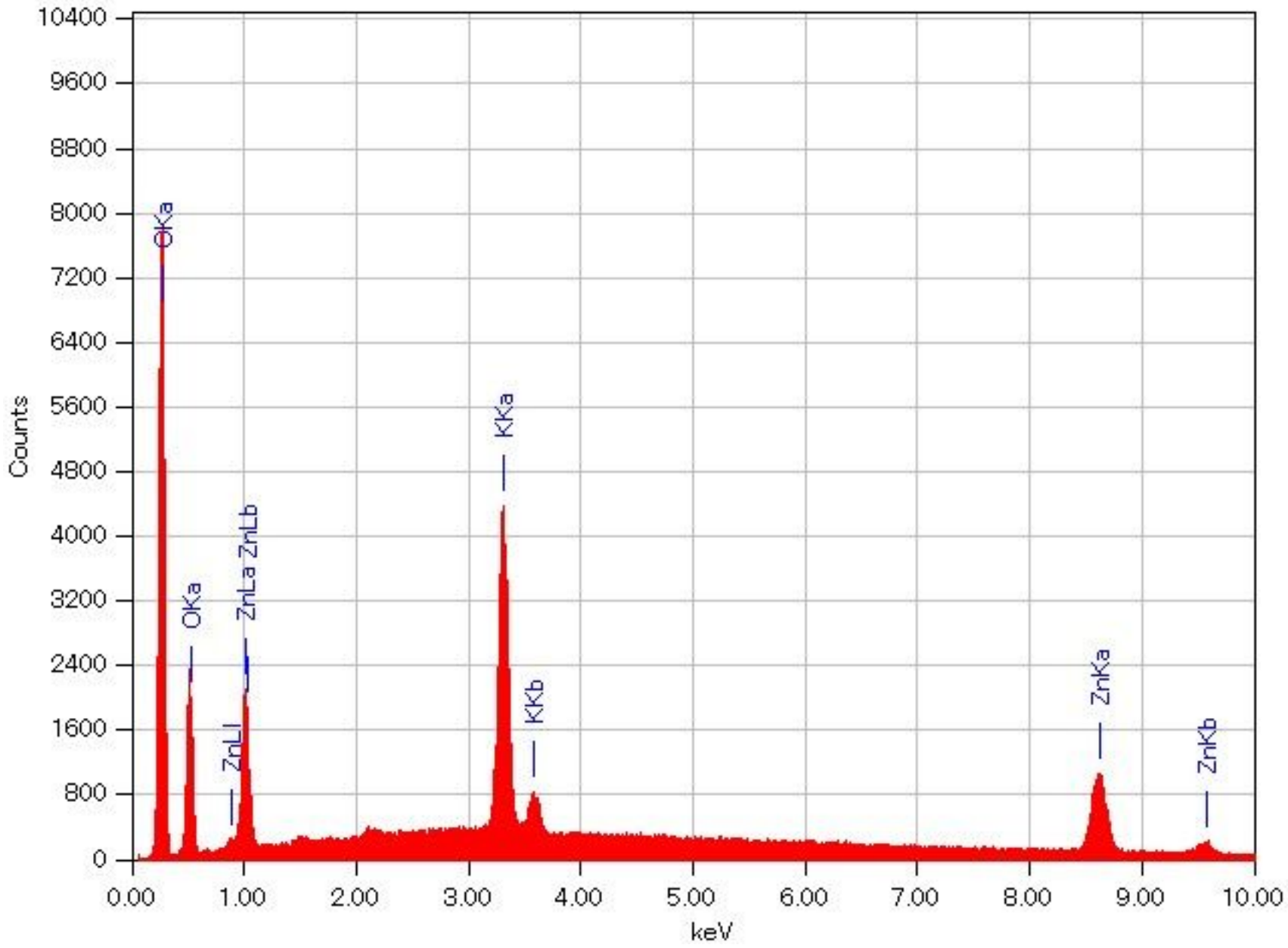

Figure S2. EDX spectra of **Zn-DEHA-K**.

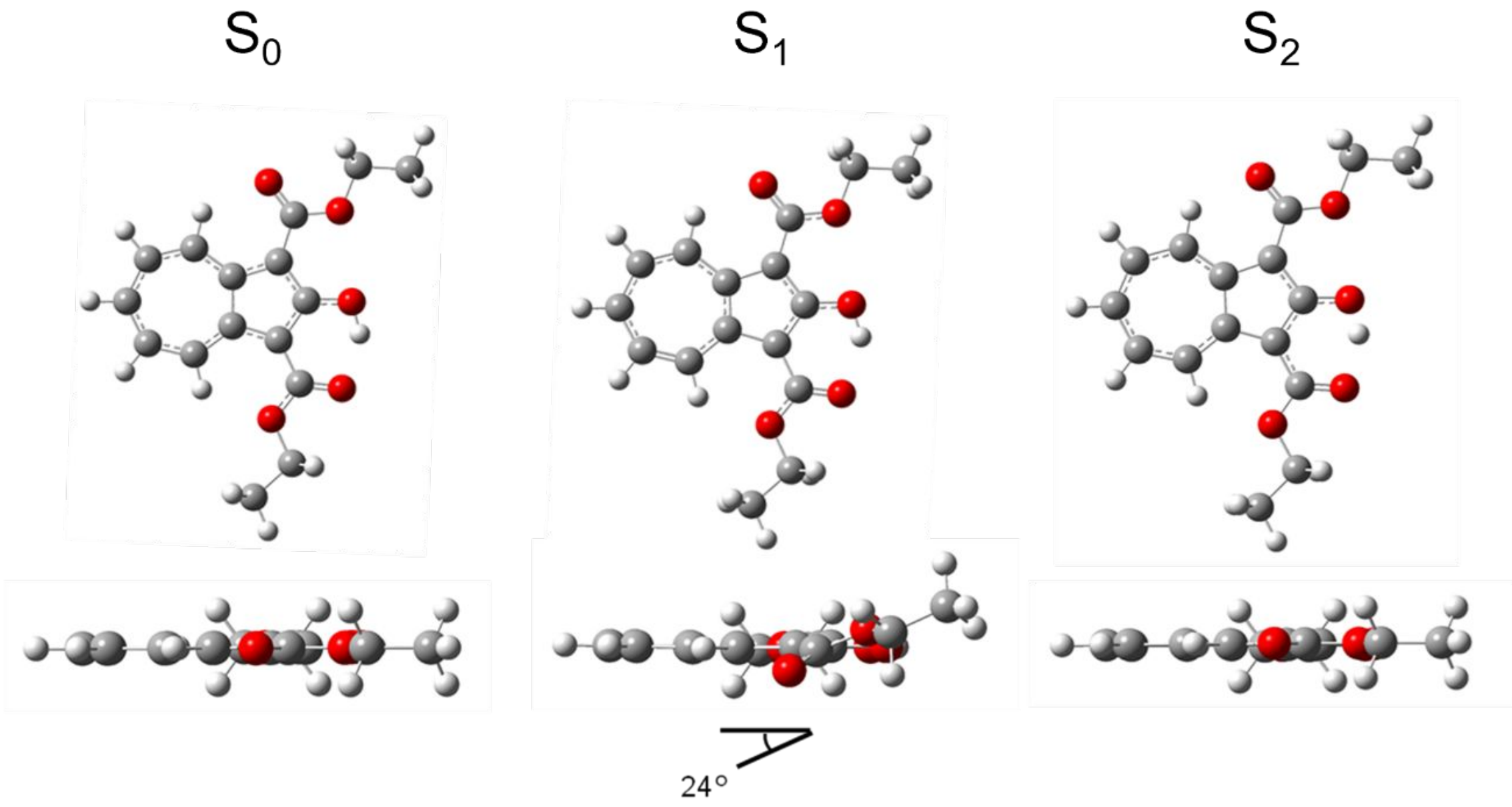

Figure S3. Optimized structure of **DEHA** in S_0, S_1, and S_2.

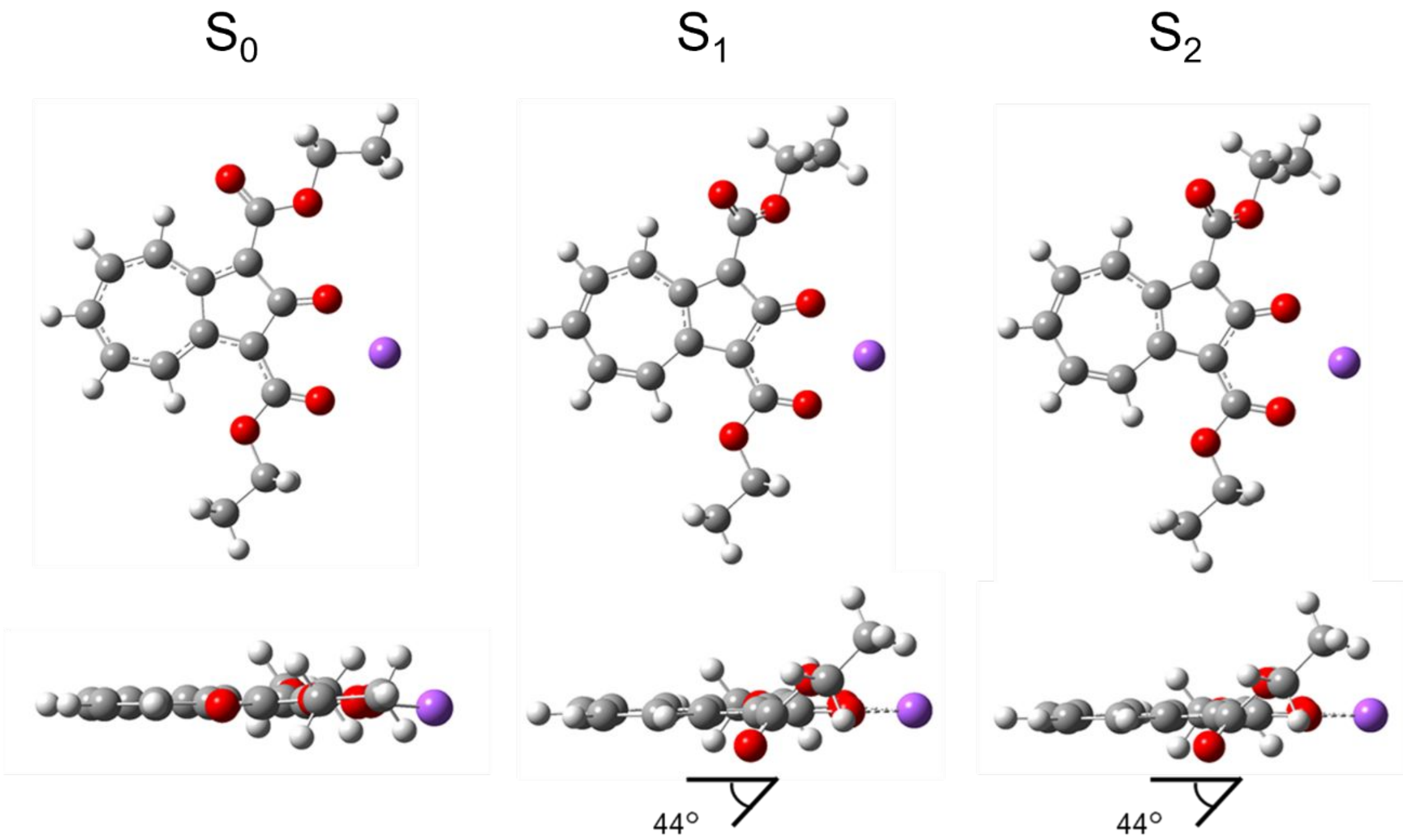

Figure S4. Optimized structure of **Na-DEHA** in S_0, S_1, and S_2.

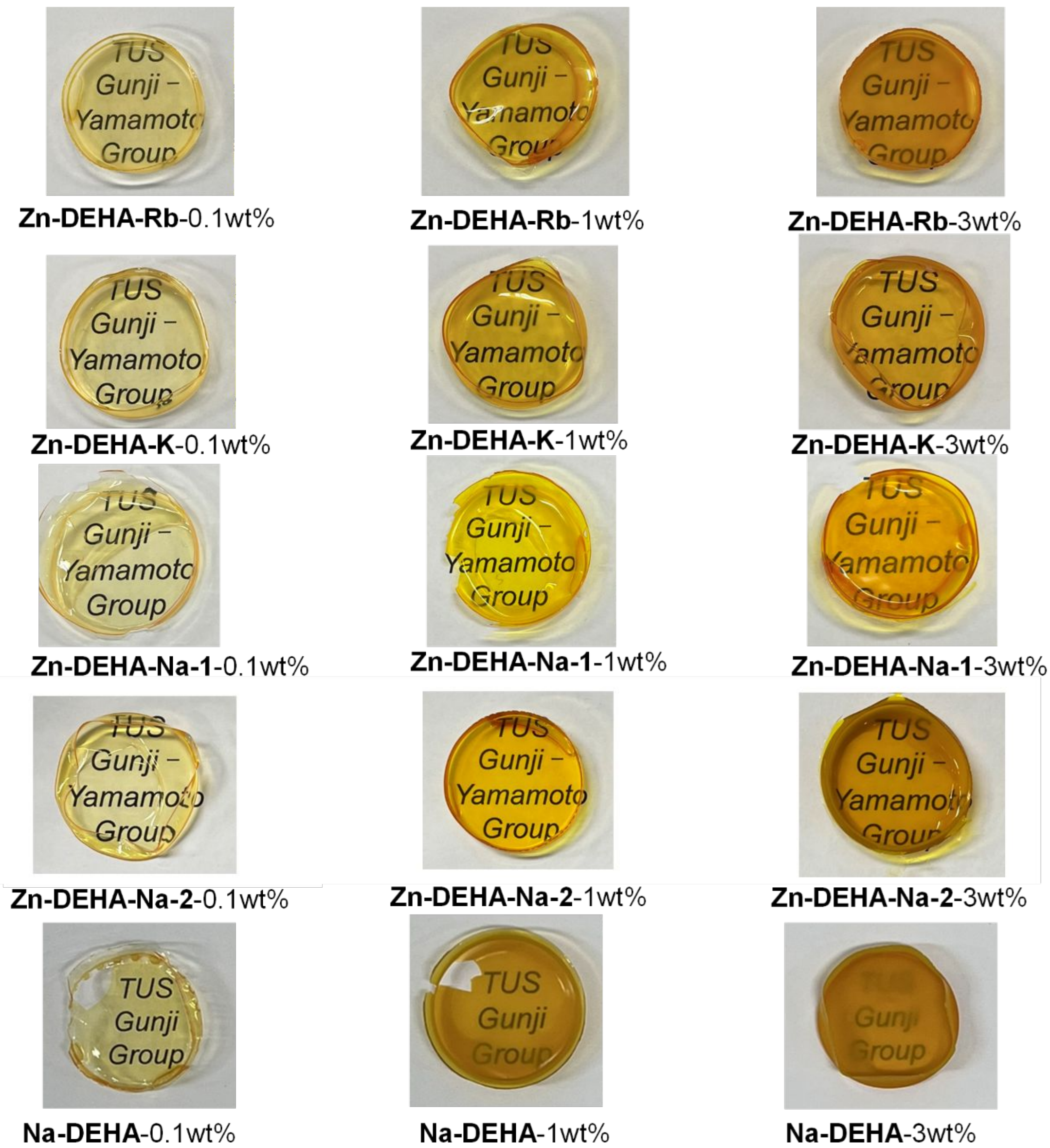

Figure S5. Appearance of PMMA composite films.

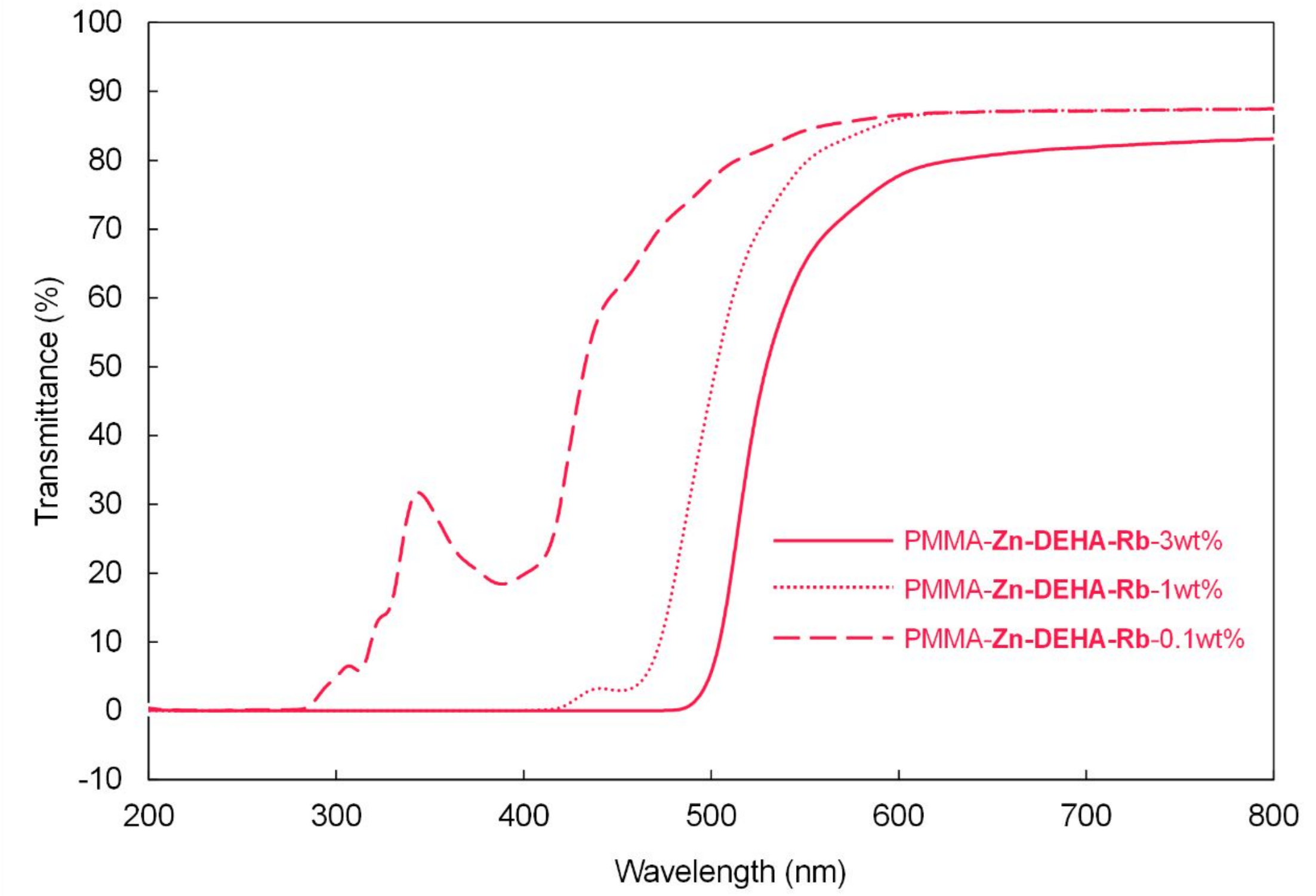

Figure S6. UV-Vis spectra of PMMA-**Zn-DEHA-Rb** composite films

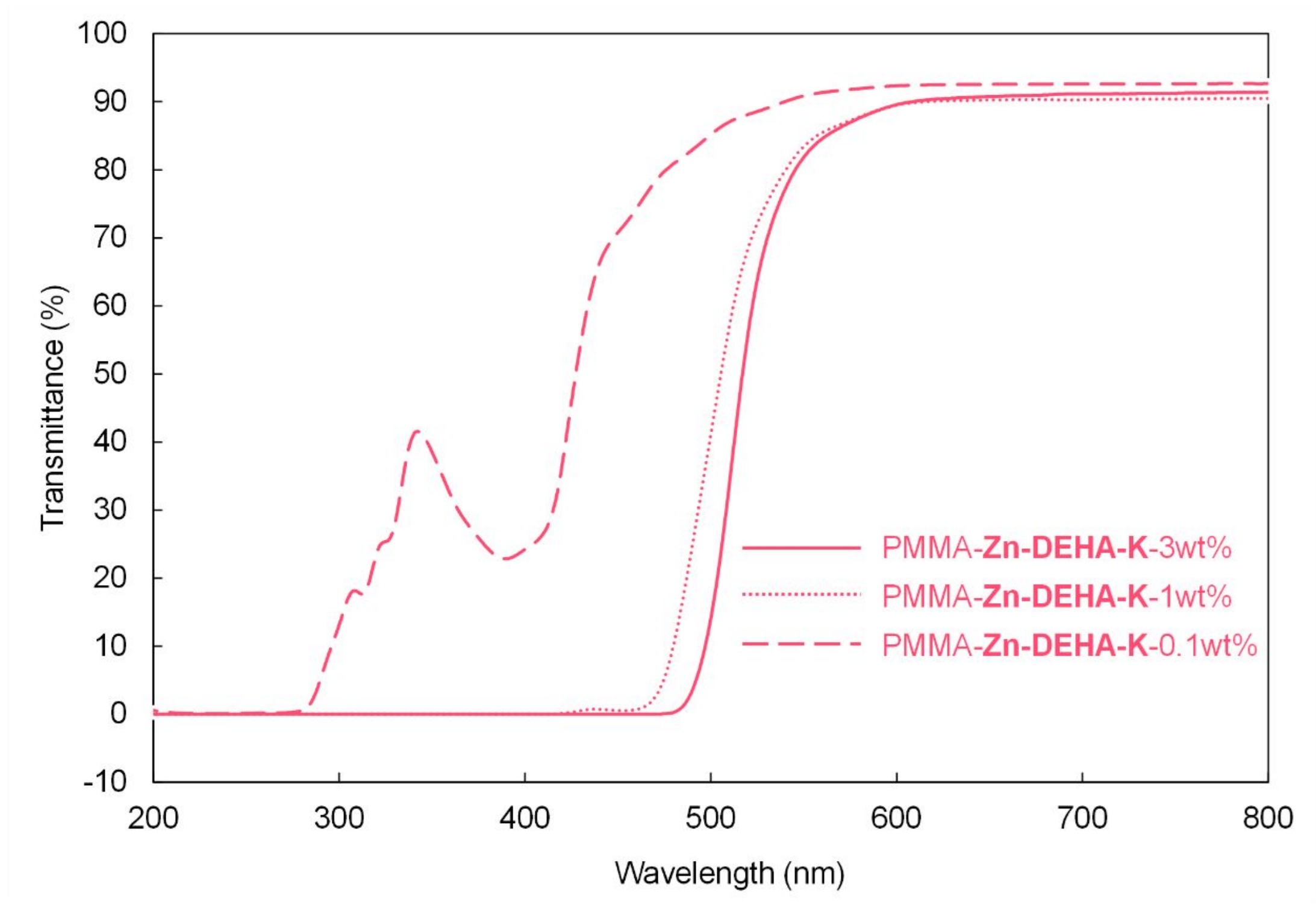

Figure S7. UV-Vis spectra of PMMA-**Zn-DEHA-K** composite films

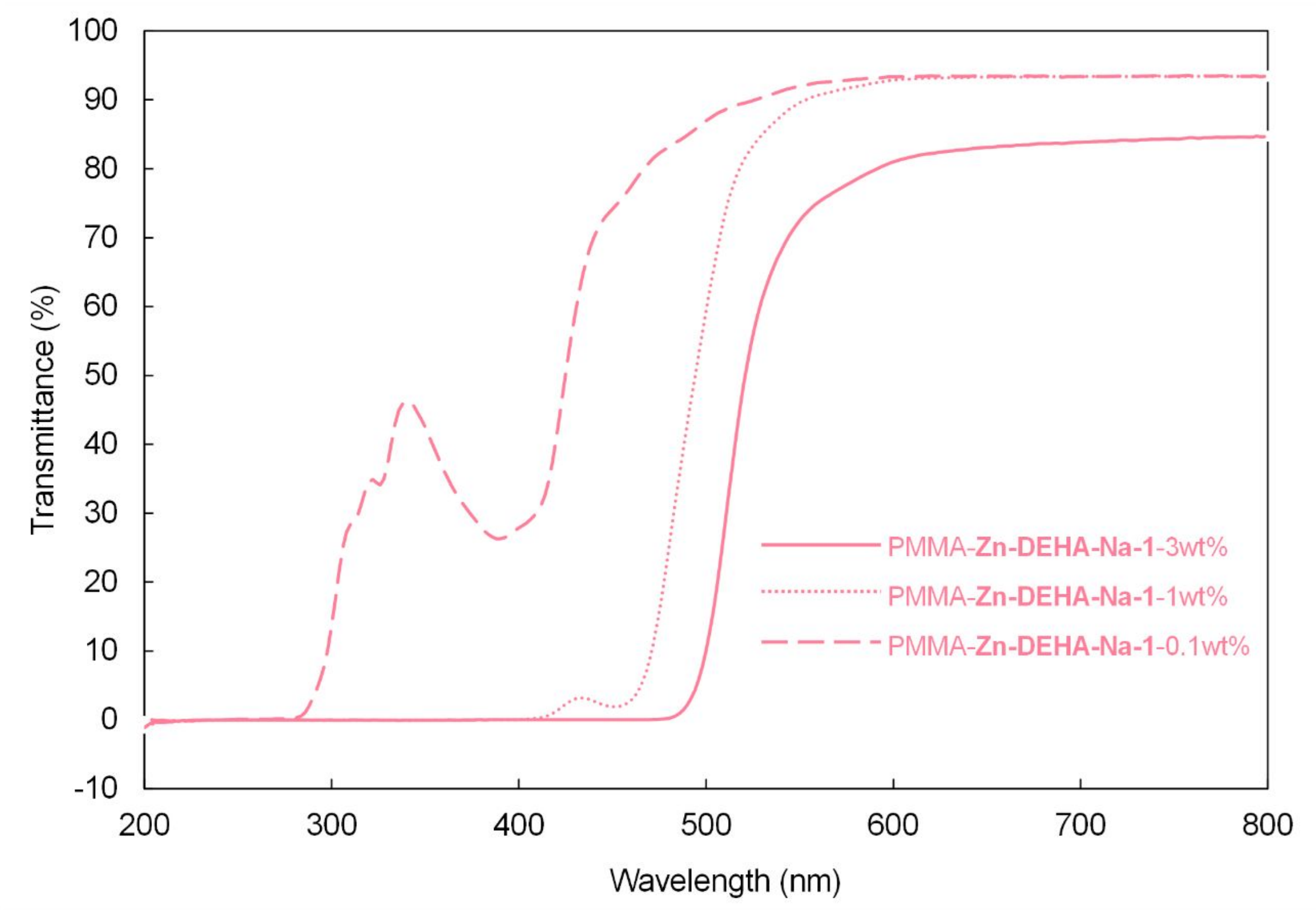

Figure S8. UV-Vis spectra of PMMA-**Zn-DEHA-Na-1** composite films

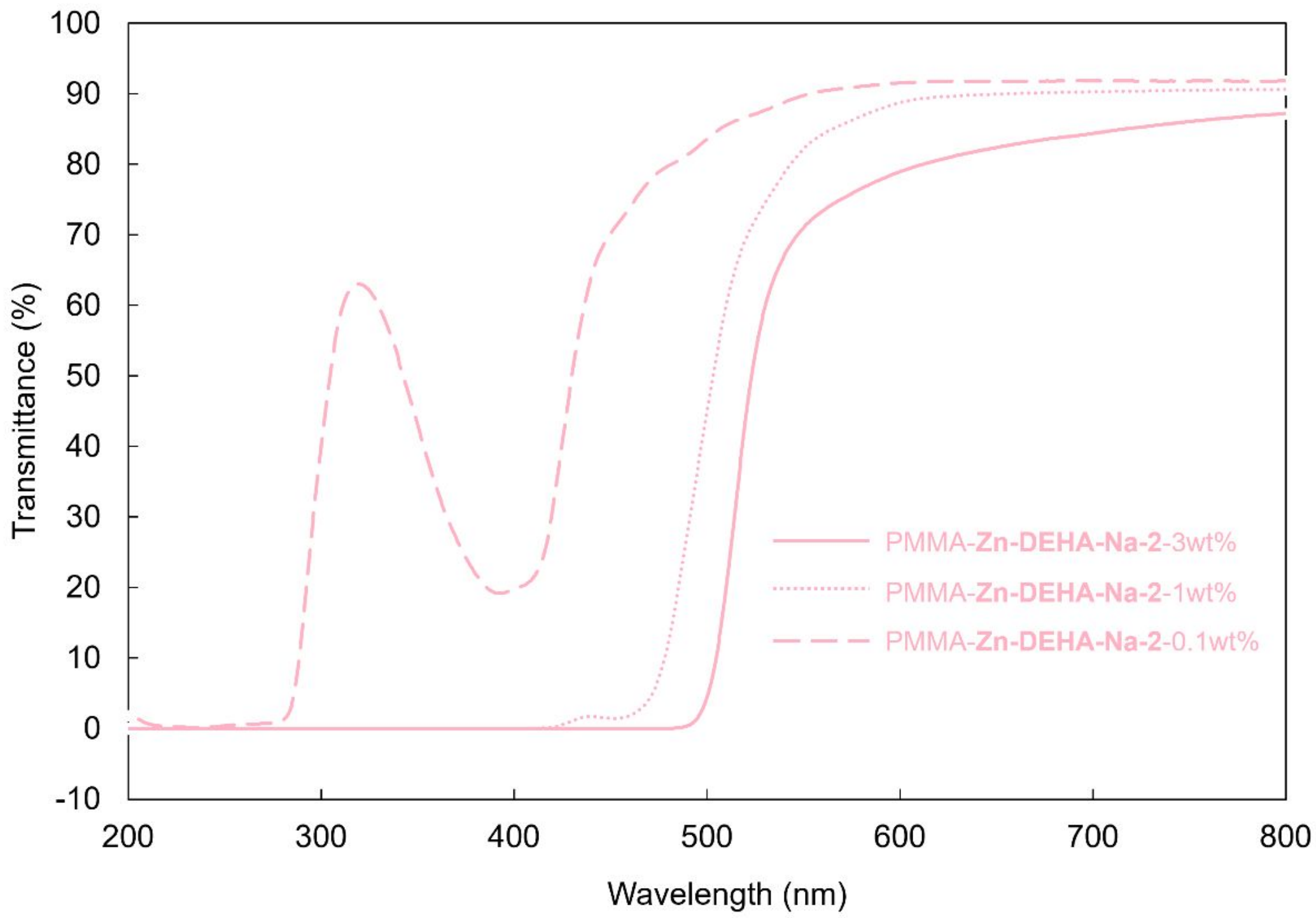

Figure S9. UV-Vis spectra of PMMA-**Zn-DEHA-Na-2** composite films

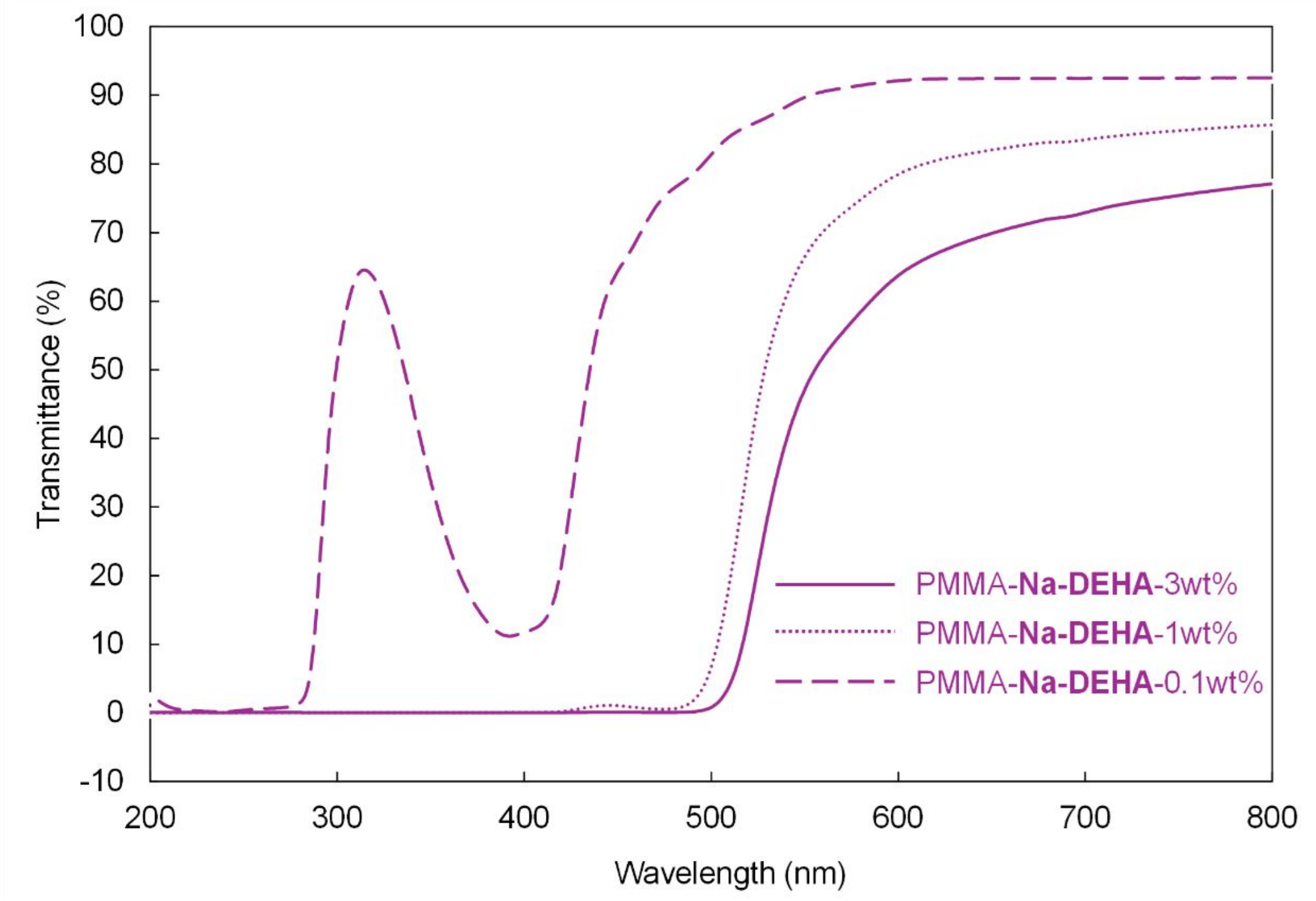

Figure S10. UV-Vis spectra of PMMA-**Na-DEHA** composite films

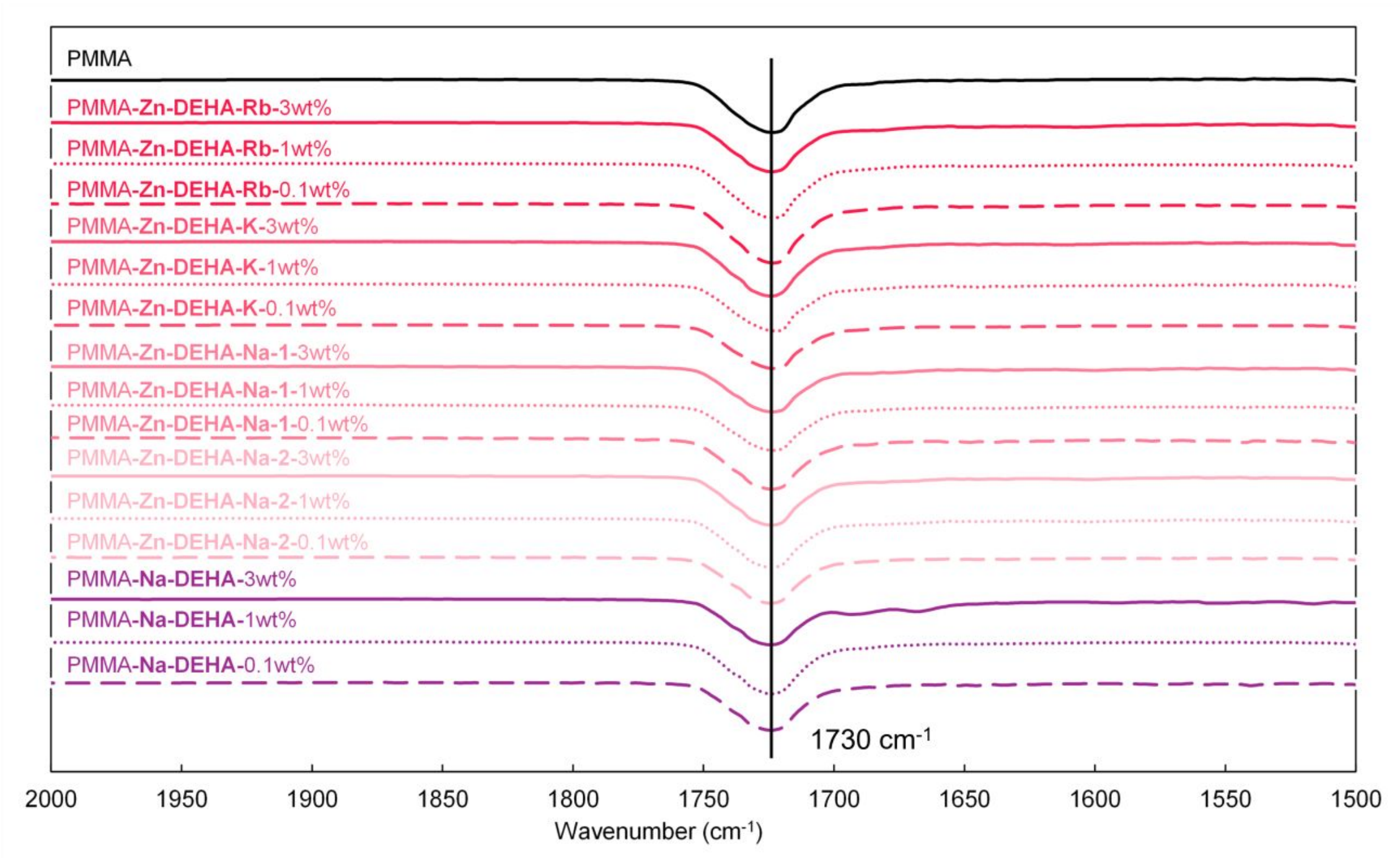

Figure S11. IR spectra of PMMA composite films (**Zn-DEHA-Rb**, **Zn-DEHA-K**, **Zn-DEHA-Na-1**, **Zn-DEHA-Na-2**, and **Na-DEHA**)

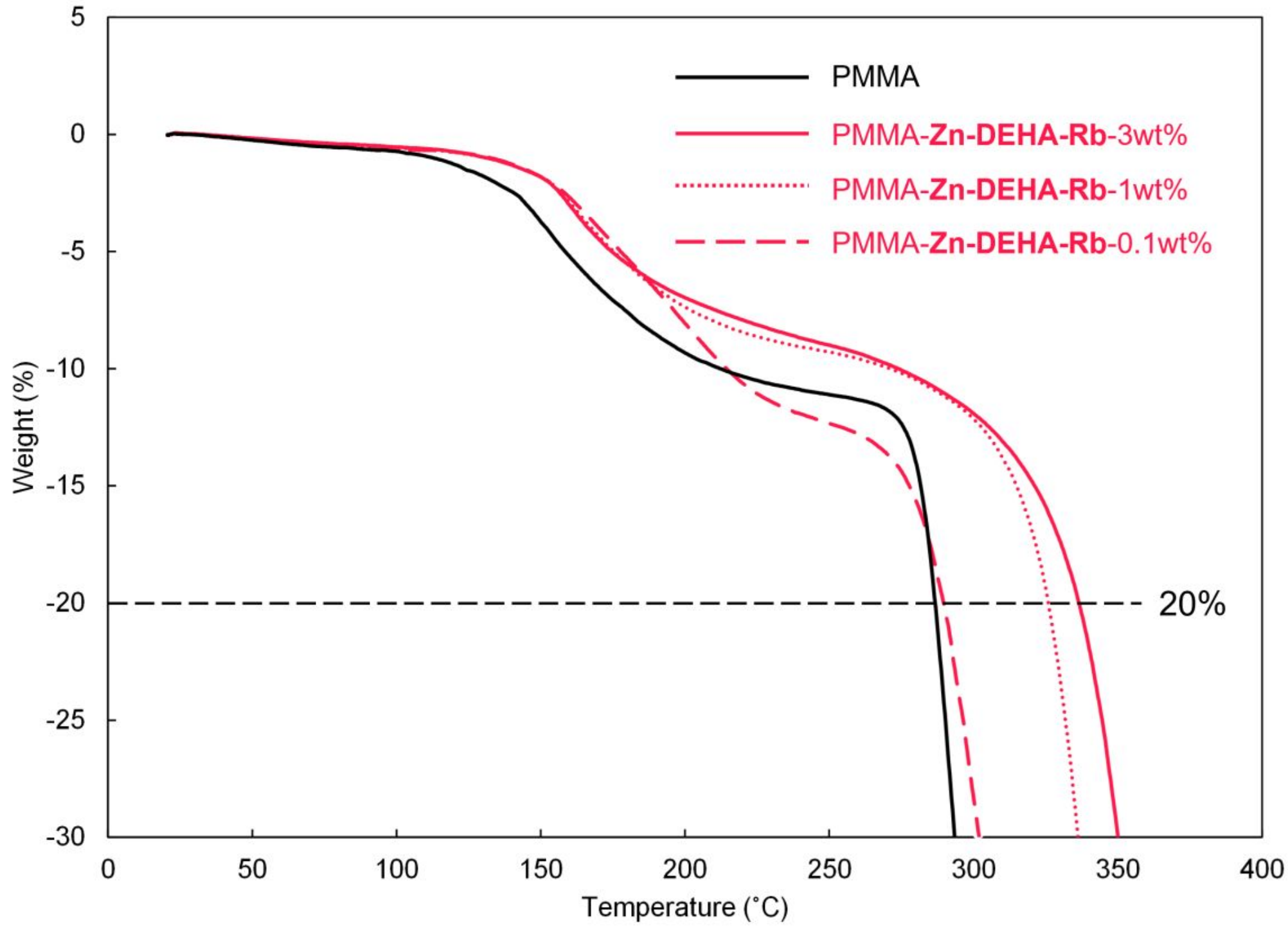

Figure S12. TG curve of PMMA-**Zn-DEHA-Rb** composite films

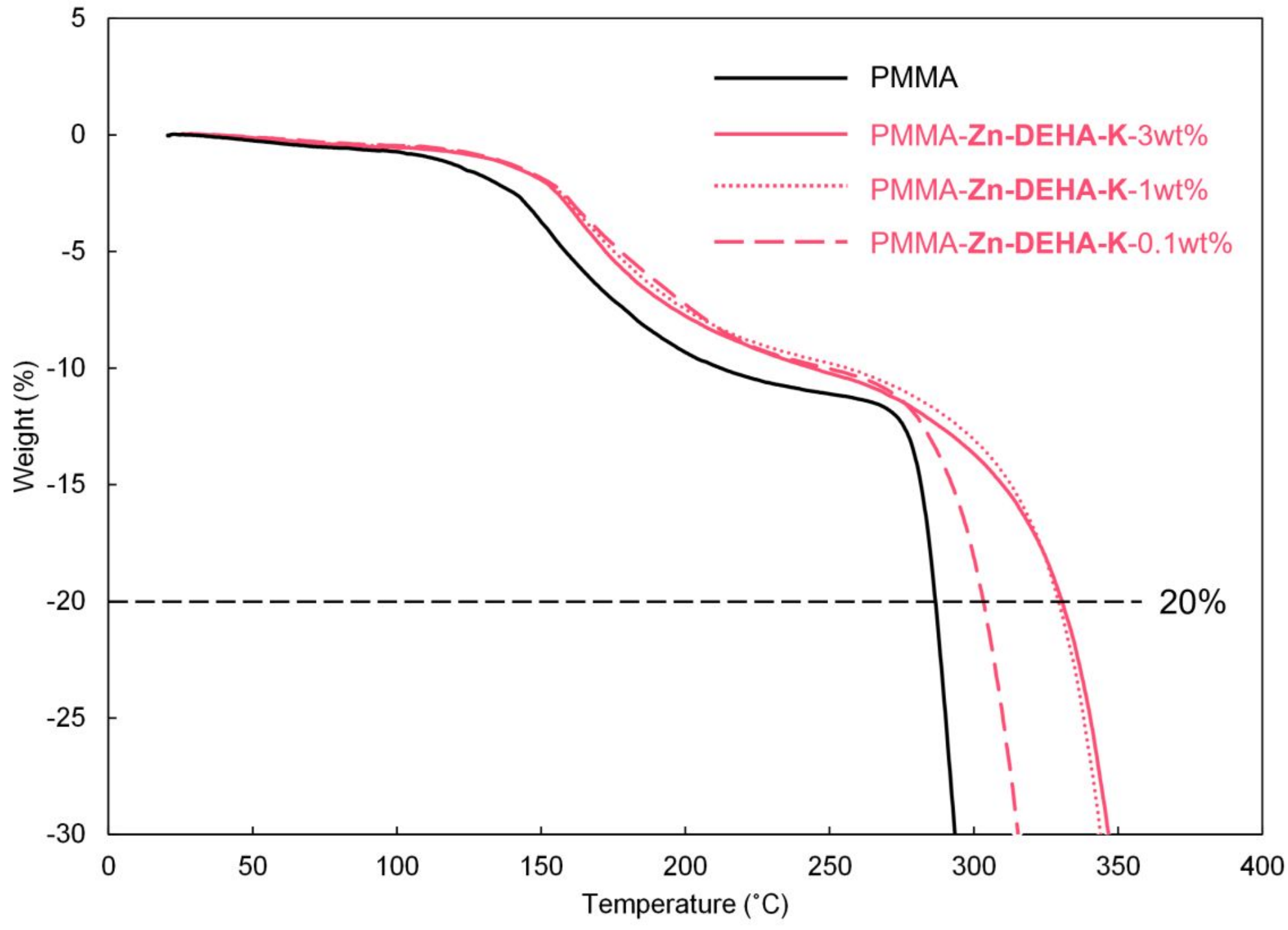

Figure S13. TG curve of PMMA-**Zn-DEHA-K** composite films

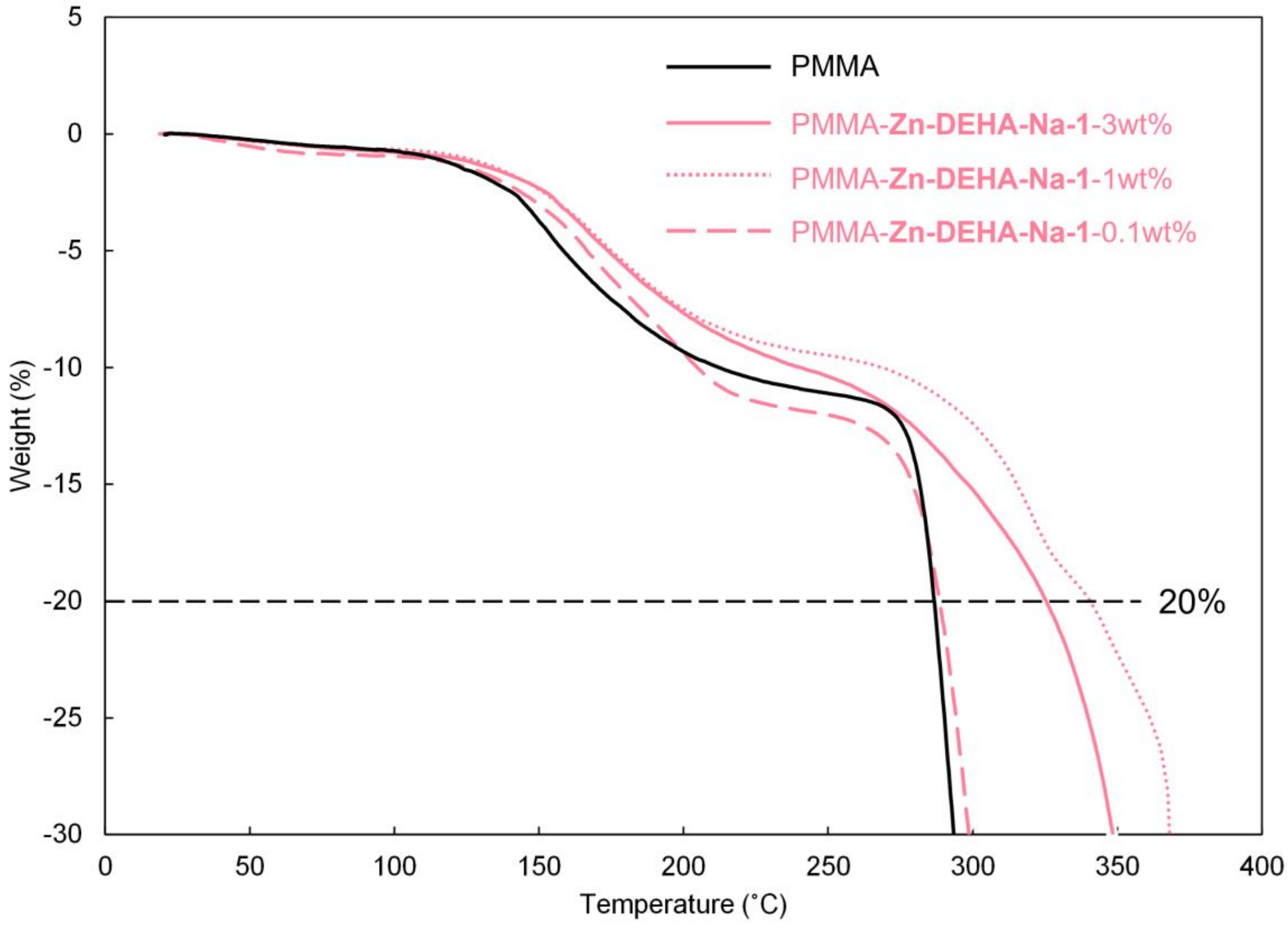

Figure S14. TG curve of PMMA-**Zn-DEHA-Na-1** composite films

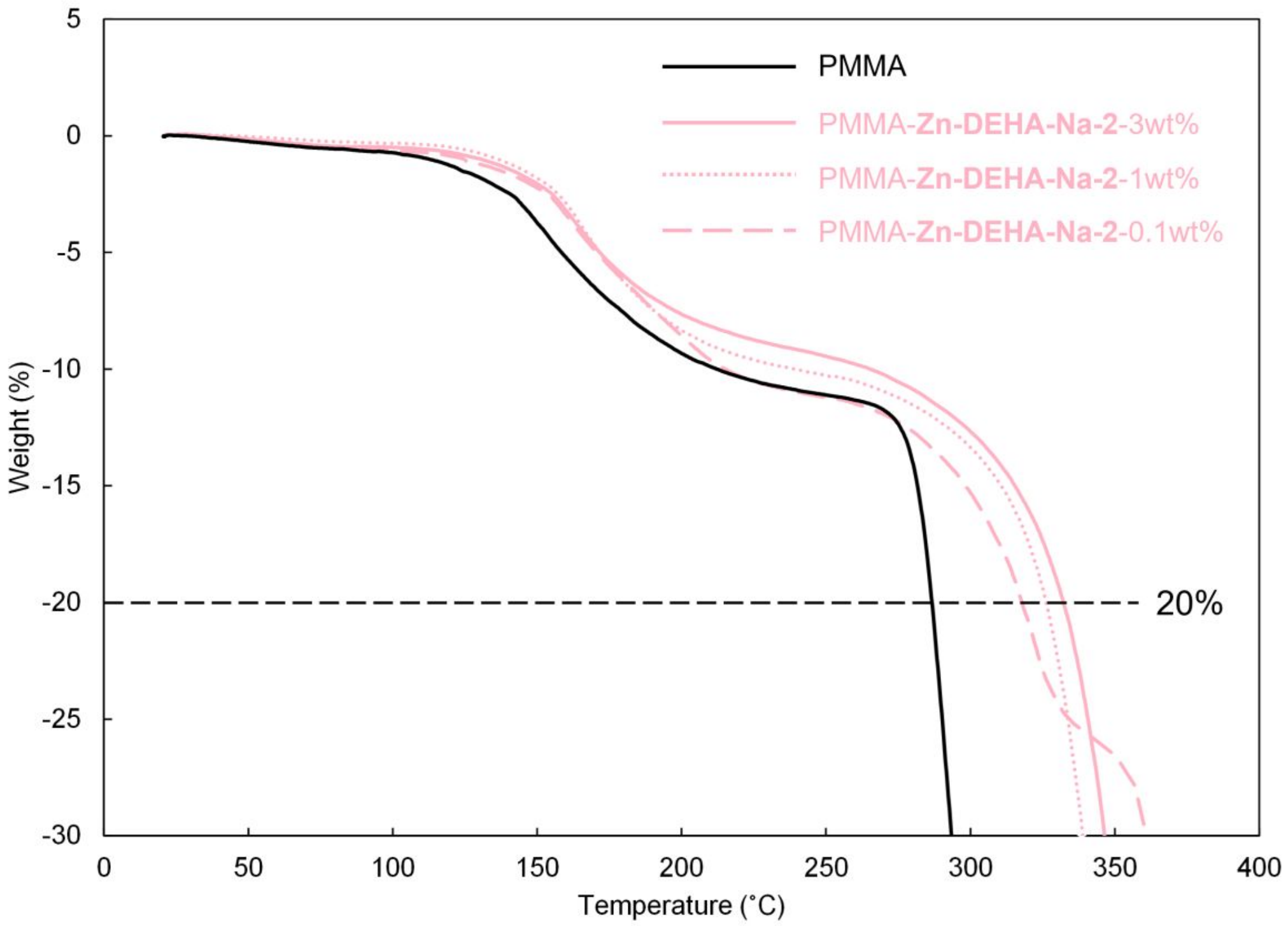

Figure S15. TG curve of PMMA-**Zn-DEHA-Na-2** composite films

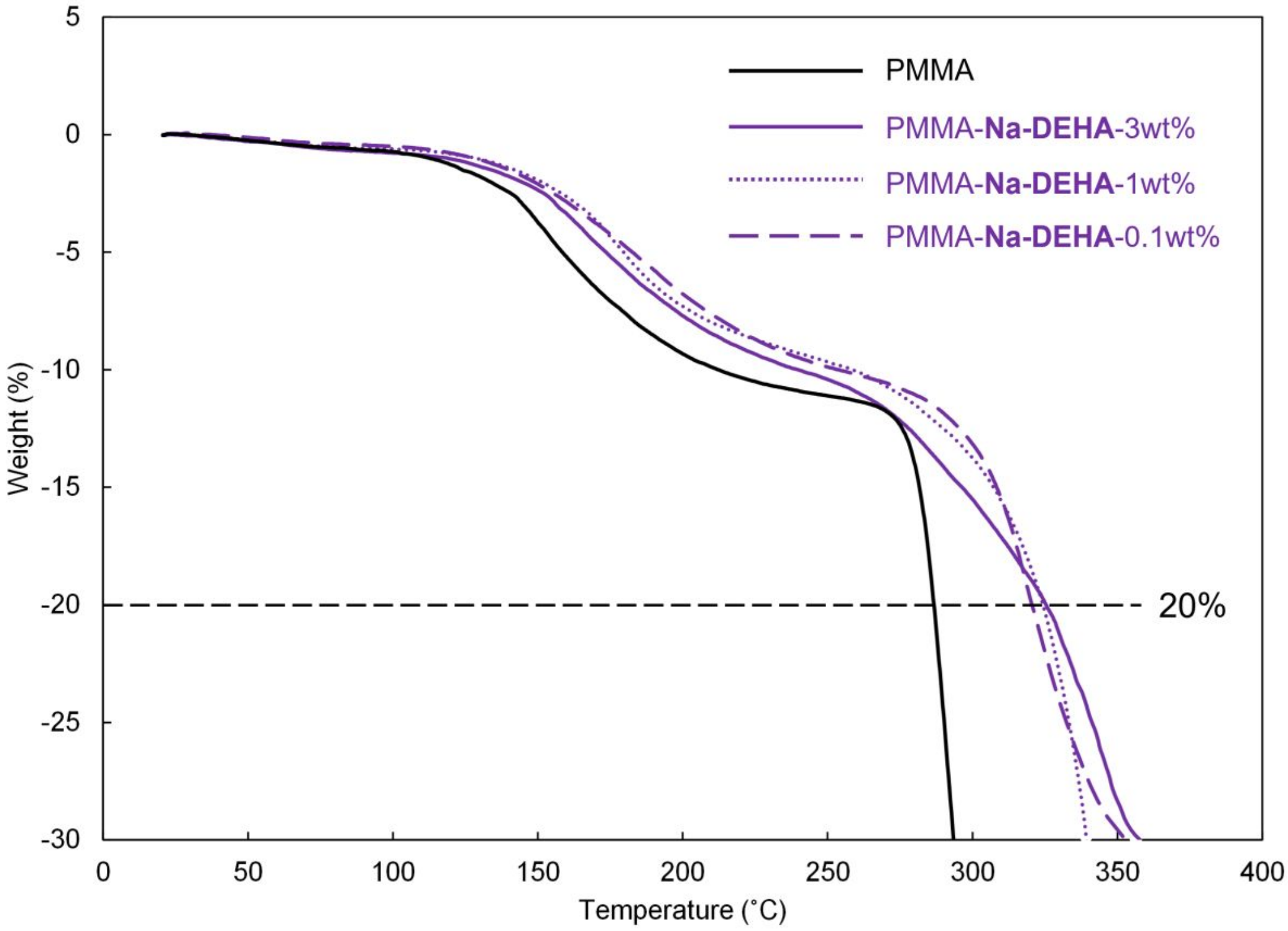

Figure S16. TG curve of PMMA-**Na-DEHA** composite films
